# Potential of Lactic Acid Bacteria as Plant-Growth-Promoting Bioinoculant Candidates with *In Vitro* Antifungal Activity: Insights from *Brassica napus* as a Model System

**DOI:** 10.3390/microorganisms14102282

**Published:** 2026-10-07

**Authors:** Haitam Lahmamsi, Samir Ananou, Zineb Ben Khadda, Nabil Radouane, Essaid Ait Barka, Tourya Sagouti, Rachid Lahlali, Said Ezrari, Abdessalem Tahiri

**Affiliations:** 1 Laboratoire de Biotechnologie Microbienne et Molécules Bioactives, Faculté des Sciences et Techniques, Université Sidi Mohamed Ben Abdellah, Route Immouzer BP 2202, Fez 30000, Morocco; haitam.lahmamsi@usmba.ac.ma (H.L.); samir.ananou@usmba.ac.ma (S.A.); 2 Phytopathology Unit, Department of Plant Protection, Ecole Nationale d’Agriculture de Meknès, Km 10, Rte Haj Kaddour, BP S/40, Meknes 50001, Morocco; atahiri@enameknes.ac.ma; 3 Laboratory for Research in Life and Drug Sciences, Faculty of Medicine and Pharmacy Oujda, University Mohammed First, BP 724 Hay Al Quods, 60000 Oujda, Morocco; benkhadda.zaynab@gmail.com (Z.B.K.); saidez@live.fr (S.E.); 4 African Genome Center, Mohammed VI Polytechnic University (UM6P), Hay Moulay Rachid, Ben Guerir 43150, Morocco; red1.nabil@gmail.com; 5 Research Unit Induced Resistance and Plant Bioprotection, USC INRAe 1488, SFR Condorcet FR CNRS 3417, Faculty of Sciences, University of Reims Champagne-Ardenne, 51100 Reims, France; 6 Laboratory of Virologie, Microbiology and Quality/Ecotoxicology and Biodiversity, Faculty of Sciences and Techniques, Hassan II University, Mohammedia 20650, Morocco; sagoutitourya@gmail.com

**Keywords:** antifungal activity, biocontrol, *Brassica napus*, dose–response, plant growth promotion

## Abstract

Lactic acid bacteria (LAB) are promising microbial candidates for sustainable agriculture, but their combined plant-growth-promoting and biocontrol potential remains underexplored. In this study, 32 previously isolated LAB strains were evaluated for *in vitro* antifungal activity against four phytopathogens, plant-growth-promoting traits, antimicrobial biosynthetic markers, safety attributes, and *in vivo* effects on *Brassica napus*. Antagonistic activity was strong but strain-dependent, with the greatest inhibition observed against *Pythium heterothallicum* and several isolates showing broad-spectrum activity against *Fusarium equiseti*, *Geotrichum citri-aurantii*, and *Botrytis cinerea*. Functional screening revealed complementary traits, including phosphate solubilization, acid phosphatase and cellulase activities, and the production of ammonia, siderophores, and hydrogen cyanide. PCR screening detected putative markers associated with iturin and subtilosin biosynthesis in a subset of strains. No hemolytic or DNase activity was detected, and no broadly concerning antibiotic resistance profiles were observed. No strain consistently outperformed all others across inoculum densities, highlighting a nonlinear dose–response relationship and the need for strain-specific inoculum optimization. Overall, several LAB strains emerged as promising dual-function bioinoculant candidates.

## 1. Introduction

In recent years, food crises have reached unprecedented levels, pushing millions of people worldwide to the brink of hunger. Approximately 295 million people experience high levels of acute food insecurity, including 2 million who face catastrophic conditions, mainly in the Gaza Strip, Sudan, South Sudan, Haiti, and Yemen [1]. Conflicts such as the ongoing war in Ukraine, one of the world’s leading cereal-producing countries, have severely disrupted global food supply chains and driven substantial price increases by restricting access to essential commodities such as maize, wheat, and sunflower oil [2]. More recently, escalating geopolitical tensions and disruptions to maritime trade through the Strait of Hormuz, a strategic corridor through which nearly 20% of global oil shipments pass, have heightened concerns about energy security and transportation costs. These disruptions have increased fuel and fertilizer prices, further exacerbating agricultural production costs, food inflation, and the vulnerability of global food systems [3].

These complex and successive crises have exposed the vulnerability of conventional agricultural systems, which often struggle to meet growing demand under conditions shaped by conflict, climate change, and resource scarcity [4]. Under these circumstances, alternatives to chemical pesticides, fertilizers, and growth enhancers are needed to reduce food losses, strengthen crop production, and promote resilient and stable agricultural productivity, particularly in food-insecure regions.

Several ecological alternatives have therefore been explored, with beneficial microorganisms receiving considerable attention as resource-efficient alternatives to chemical inputs. Often referred to as plant-growth-promoting microorganisms (PGPMs), effective microorganisms, or biofertilizers, these microbes use several mechanisms to enhance plant growth and stress tolerance, including phosphate solubilization, organic matter decomposition, phytohormone and lytic enzyme secretion, siderophore production, and the induction of plant defenses [5].

Microbial inoculants can stimulate plant defense mechanisms, enhance nutrient uptake, improve soil structure, and help preserve ecological balance in crop production systems [6,7]. Unlike chemical inputs, these biological agents can contribute to long-term soil stability and fertility through synergistic interactions with the plant rhizosphere and surrounding soil. Their use aligns with the principles of sustainable intensification, which seeks to increase crop yields without depleting natural resources or harming the environment [8].

Because of their multifunctional roles, beneficial bacteria have become important components of sustainable agriculture and offer environmentally sound alternatives to synthetic chemicals. Several bacterial taxa have shown considerable potential for plant growth promotion and disease suppression, including *Rhizobium*, *Bacillus*, *Pseudomonas*, and *Azospirillum* species. However, several constraints limit their adoption in large-scale agricultural systems. The efficacy of bacterial inoculants is often influenced by soil type, the native microbial community, and climatic conditions, all of which affect their survival, adaptation, functional stability, and colonization under field conditions [9,10].

In this context, lactic acid bacteria (LAB) are a promising but underused group of candidate inoculants for sustainable agriculture. Traditionally used as starter cultures in food fermentation and preservation, LAB have gained broad consumer acceptance because many taxa have Qualified Presumption of Safety (QPS) status in the European Union or Generally Recognized as Safe (GRAS) status under the US FDA framework [11]. Unlike many other PGPMs, LAB can adapt to a wide range of natural niches, including the phyllosphere, rhizosphere, plant surfaces, and internal plant tissues [12].

Their broad repertoire of metabolites—organic acids, enzymes, and antimicrobial compounds—makes LAB promising candidates for use as biofertilizers, biocontrol agents, and plant growth promoters. LAB also tolerate several environmental stresses, including fluctuations in temperature, acidity, and oxidative conditions, and their resilience has been documented across multiple species and ecological niches [13]. This tolerance may allow LAB to remain active under conditions that adversely affect conventional plant-growth-promoting rhizobacteria. In addition, LAB may strengthen plant defenses and stress-mitigation mechanisms through the secretion of exopolysaccharides, organic acids, compatible solutes, and other bioactive or antioxidant-inducing metabolites [14,15]. Their capacity both to tolerate stress and to mitigate its effects supports their potential to promote plant growth, particularly in regions with highly variable environmental conditions.

Given the growing interest in resilient and consumer-acceptable microbial solutions for sustainable agriculture, this study aimed to evaluate the *in vitro* antagonistic activity of 32 previously isolated and identified LAB strains against major fungal and oomycete pathogens, characterize their plant-growth-promoting biochemical traits, and assess their *in vivo* efficacy using *Brassica napus* as a model plant under controlled conditions. This integrated approach was used to characterize the functional capabilities of the LAB strains and evaluate their suitability as environmentally sound inputs for crop production systems.

## 2. Materials and Methods

### 2.1. Strains and Biological Materials

In this study, 32 previously isolated, purified, and identified LAB strains were tested for antagonistic activity against major fungal and oomycete pathogens and assessed for plant-growth-promoting activity. The collection comprised strains of *Lactiplantibacillus plantarum* and *Levilactobacillus brevis*, *Lactiplantibacillus pentosus*, *Lacticaseibacillus rhamnosus* and one strain of *Enterococcus durans* (Table S1).

*In vivo* assays were conducted using canola seeds (*Brassica napus*) provided by the Plant Pathology Unit at the National School of Agriculture, Meknes, Morocco. The phytopathogenic species used as models (*Geotrichum citri-aurantii*, *Pythium heterothallicum*, *Botrytis cinerea*, and *Fusarium equiseti*) were obtained from the fungal culture collection of the same institution (Table S2).

### 2.2. In Vitro Antagonism Against Fungal Pathogens

After revival from glycerol stocks, the 32 LAB strains were grown in de Man, Rogosa, and Sharpe (MRS) broth (Merck, Darmstadt, Germany) and subcultured on MRS agar for 48 h at 37 °C. Antagonistic activity against the pathogens was assessed using a dual-culture assay in which bacterial strains were streaked opposite the pathogen mycelium. Four equidistant streaks of each LAB strain were made at the periphery of potato dextrose agar (PDA) plates (Merck, Germany), 1 mm from the edge, and the plates were incubated for 2 h at 37 °C. A 5 mm mycelial plug from each pathogen was then placed at the center of a plate. Plates containing only a pathogen plug served as negative controls. All plates were incubated at 25 °C until the mycelium in the control plates reached the plate margin. Each treatment was performed in triplicate, and the experiment was independently repeated twice.

After incubation, mycelial growth was measured with a caliper, and the inhibition rate was calculated using the formula described by Legrifi et al. (2025) [16]:

IR = [((Diameter of the fungal control − Diameter of the fungal colony in the test))/(Diameter of the fungal control)] × 100.

### 2.3. Effect of pH Neutralization on Antimicrobial Activity

To assess the contribution of organic-acid-mediated acidification to the antifungal activity of this collection of LAB strains, the direct confrontation assay between the LAB strains and the 4 pathogens was repeated using agar medium buffered to pH 7.0. Under these conditions, the inhibitory effect of acidic metabolites was neutralized, and loss of inhibitory activity was interpreted as evidence that the antimicrobial activity was mainly associated with acidification rather than pH-independent antimicrobial metabolites.

### 2.4. Assessment of In Vitro PGP Traits

To identify the most effective strains, a series of *in vitro* assays was conducted to characterize key plant-growth-promoting (PGP) traits of the LAB strains, including phosphate solubilization; acid and alkaline phosphatase activities; cellulase and other hydrolytic enzyme activities; indole-3-acetic acid (IAA), siderophore, ammonia, and hydrogen cyanide production; and nitrogen fixation.

#### 2.4.1. Protease Activity

The proteolytic activity of the LAB strains was assessed using skim milk agar (SMA), as described by Abubakr and Al-Adiwish (2017) [17]. Briefly, 500 mL of a 2.5% agar solution was sterilized at 121 °C for 20 min. Separately, 25 g of skim milk powder was suspended in 250 mL of distilled water and sterilized at 110 °C for 10 min. The solutions were cooled to approximately 50 °C, thoroughly mixed, and poured into plates. A LAB suspension containing 1 × 10^8^ CFU/mL was prepared from fresh cultures, and 5 µL of each suspension was spotted onto SMA plates. The plates were incubated at 30 °C for 48 h. Clear hydrolysis zones around the colonies indicated proteolytic activity. The proteolytic index was calculated as the ratio of the diameter of the colony plus the halo to the colony diameter, as described by Syed Ab Rahman et al. (2018) [18].

#### 2.4.2. Phosphate Solubilization

The ability of the isolates to solubilize tricalcium phosphate, an insoluble form of phosphate, was assessed on Pikovskaya medium as described previously [16,19]. A 5 µL aliquot of freshly prepared bacterial suspension (1 × 10^8^ CFU/mL) was spotted onto Pikovskaya agar, and the plates were incubated at 30 °C for 5 days. After incubation, the diameters of the solubilization halo and colony were measured. The phosphate solubilization index was calculated using the formula described by Syed Ab Rahman et al. (2018) [18].

#### 2.4.3. Alkaline and Acid Phosphatase Activities

Phosphatase activities in LAB were determined using the p-nitrophenyl phosphate (pNPP) assay with slight modifications [19,20]. Briefly, 48 h LAB liquid cultures were prepared. Bacterial cells were harvested by centrifugation at 10,000× *g* for 20 min and washed with saline solution (0.9%). The cells were resuspended in 500 µL of Tris buffer (pH 7.0) and sonicated at 4 °C using alternating 1 min sonication and 1 min rest periods. Cell debris was removed by centrifugation at 10,000× *g* for 5 min at 4 °C, and the supernatant was retained for analysis.

Phosphatase activity was determined by mixing 250 µL of the enzymatic extract with an equal volume of either acid phosphatase buffer [5.5 mM pNPP in 0.05 M citrate buffer (pH 4.8)] or alkaline phosphatase buffer [5.5 mM pNPP in 0.05 M glycine buffer containing 0.01% MgCl_2_·6H_2_O (pH 10.5)] in microplates. The plates were sealed and incubated at 37 °C for 30 min. The reaction was stopped by adding 0.5 N NaOH, and absorbance was measured at 405 nm using a microplate reader (Multiskan FC, Thermo Fisher Scientific, Vantaa, Finland). Absorbance values were converted to phosphatase activity (µmol h^−1^) using a p-nitrophenol standard curve.

#### 2.4.4. Pectinase Activity

Pectinolytic activity was determined according to Mohd Jaini et al. (2022) [21]. A basal medium containing yeast extract (5 g), peptone (5 g), K_2_HPO_4_ (1 g), MgSO_4_·7H_2_O (0.2 g), and agar (15 g) [22], supplemented with 1% apple pectin, was used. After the sterile medium was poured into Petri dishes, two equidistant wells were made with a cork borer. Each well was filled with 100 µL of a 24 h LAB culture, and the plates were incubated at 37 °C for 3 days.

After incubation, pectinase activity was revealed by flooding the plates with iodine solution. A positive reaction was indicated by the formation of clear halos around the wells. For positive reactions, the activity was quantified using the formula described above [18].

#### 2.4.5. Siderophore Production

The ability of the LAB strains to produce siderophores was assessed using the chrome azurol S (CAS) shuttle assay, first described by Schwyn and Neilands (1987) [23], with slight modifications.

The CAS solution was prepared as follows. In a 100 mL volumetric flask, 6 mL of hexadecyltrimethylammonium bromide solution (HDTMA, 10 mM) was mixed with sterile distilled water. Separately, a fresh iron(III) solution was prepared by dissolving FeCl_3_·6H_2_O in 10 mM HCl to a final concentration of 1 mM. A 1.5 mL aliquot of this solution was mixed with 2 mM CAS solution under magnetic stirring. Piperazine buffer was prepared by dissolving 4.307 g of anhydrous piperazine in sterile distilled water and adding 6.25 mL of 12 M HCl to obtain a pH of 5.6. The buffer was transferred to the 100 mL flask, and the volume was adjusted to 100 mL with sterile distilled water [24].

The LAB strains were inoculated in 5 mL AT minimal broth as described by Charest et al. (2005) [25] and incubated for 3 days with shaking (150 rpm). After incubation, bacterial cells were removed by centrifugation, and 0.5 mL of the supernatant was added to an equal volume of the CAS-shuttle solution and incubated for 20 min in the dark. A tube containing uninoculated AT medium was used as the control. A color change from blue to violet or orange indicated the presence of siderophores [25].

After incubation in the dark, absorbance was measured at 630 nm using a spectrophotometer (NanoDrop One/OneC, Thermo Fisher Scientific, Vantaa, Finland ). Siderophore production was expressed as percent siderophore units (PSU) and calculated using the formula described previously [26]:

$$\mathrm{Siderophore}\;\mathrm{production}:\;(\mathrm{psu})=\frac{\left(\mathrm{Ar}-\mathrm{As}\right)\times 100}{\mathrm{Ar}}$$

where Ar is the absorbance of the reference (control) and As is the absorbance of the sample (strain supernatant) [27].

#### 2.4.6. Evaluation of Indole-3-Acetic Acid Production

Indole-3-acetic acid (IAA) is a common naturally occurring auxin. The ability of the LAB strains to produce IAA was assessed using the Salkowski colorimetric method. Liquid cultures were prepared in MRS broth and incubated for 3 days at 37 °C. The cultures were centrifuged, and the pellets were discarded. One volume of supernatant was mixed with two volumes of Salkowski reagent prepared by dissolving 1.2 g of FeCl_3_ in 100 mL of 7.9 M H_2_SO_4_ [28]. A color change from yellow to pink indicated a positive reaction, which was quantified as described previously [21].

#### 2.4.7. Ammonia Production

Ammonia production was assessed according to Cappuccino and Sherman (1996) and Mohd Jaini et al. (2022) [21,29]. Sterile glass tubes containing 10 mL of peptone water were inoculated with 20 µL of each LAB culture and incubated for 24 h with shaking at 150 rpm. After incubation, 0.5 N Nessler’s reagent (Sigma-Aldrich, Taufkirchen, Germany) was added. The development of a yellow-to-brown color indicated ammonia production, and relative levels were initially estimated from color intensity. For quantification, optical density was measured using a spectrophotometer (NanoDrop One/OneC, Thermo Fisher Scientific, Vantaa, Finland ) at λ = 450 nm, and ammonia concentration was calculated from a standard curve prepared using known concentrations of ammonium sulfate [21].

#### 2.4.8. Amylase

Amylase activity was assessed using a cornstarch-based medium. Two equidistant 5 µL aliquots of bacterial suspension (10^8^ CFU mL^−1^) from each strain were spotted onto each plate, and the plates were incubated at 37 °C for 72 h. After incubation, the plates were flooded with iodine solution for 3 min and rinsed with tap water. Clear halos around bacterial spots indicated amylase activity, and the amylolytic index was calculated using the formula described above [18].

#### 2.4.9. Hydrogen Cyanide Production (HCN)

Hydrogen cyanide production was assessed using a modified version of the protocol described by Rijavec and Lapanje (2016) [30]. Each LAB isolate was grown in MRS broth (Merck, Germany) for 24 h, after which 100 µL was spread onto Bennett agar supplemented with 4.4 g L^−1^ glycine [31]. The lid of each plate was lined with Whatman filter paper that had been immersed for 1 min in a solution containing 2% sodium carbonate and 0.5% picric acid. *Pseudomonas koreensis* was used as a positive control. The plates were incubated inverted at 30 °C for 3 days. A color change from yellow to orange or brown indicated a positive result. HCN production was classified semiquantitatively as weak (+), moderate (++), strong (+++), or undetectable (-).

#### 2.4.10. Nitrogen Fixation

The ability of LAB strains to fix atmospheric nitrogen was evaluated using Ashby’s agar containing mannitol (10 g), MgSO_4_·7H_2_O (0.2 g), KH_2_PO_4_ (0.2 g), CaSO_4_·2H_2_O (0.1 g), CaCO_3_ (5 g), NaCl (0.2 g), agar (18 g), and distilled water (1 L), adjusted to pH 7.0–7.5 [32]. The plates were incubated at 30 °C for 7 days. Strains that grew on this medium were considered capable of fixing atmospheric nitrogen.

### 2.5. PCR Detection of Lipopeptide- and Bacteriocin-Associated Genes

Genomic DNA was extracted from overnight LAB cultures using the MagListo 5M Genomic DNA Extraction Kit (Bioneer, Daejeon, Republic of Korea) according to the manufacturer’s instructions. The resulting DNA was used as a template for PCR detection of biosynthetic genes associated with the antimicrobial lipopeptides bacillomycin, fengycin, iturin, surfactin, and subtilin, using the primer pairs BACC1F/BACC1R, FEND1F/FEND1R, ITUP1F/ITUP2R, P17/P18, and SpaS_F/SpaS_R, respectively. The bacteriocin-associated subtilosin gene was screened using the primer pair oSboP1N/oSboP2N (Table S3).

All PCR assays were performed using DreamTaq Hot Start Green DNA Polymerase (Thermo Scientific, Waltham, MA, USA ) in a final volume of 25 µL. Each reaction contained 0.1 µL of DreamTaq polymerase, 1 µL of each primer, 0.5 µL of dNTP mixture, 2.5 µL of DreamTaq buffer, 2.5 µL of template DNA, and 17.4 µL of nuclease-free water. A no-template control was included. Amplification was performed in an Applied Biosystems ProFlex PCR system (Thermo Scientific, Waltham, MA, USA) with initial denaturation at 95 °C for 3 min, followed by 35 cycles of denaturation at 95 °C for 30 s, annealing at the temperature specified in Table S3, and extension at 72 °C for 1 min, with a final extension at 72 °C for 10 min. PCR products were separated on 1% agarose gels stained with SYBR Safe DNA Gel Stain (Invitrogen, Carlsbad, CA, États-Unis) and visualized using an iBright CL750 Imaging System (Thermo Scientific, Waltham, MA, USA).

### 2.6. Safety Assessment of LAB Strains

To assess the safety of the LAB strains for prospective applications and human consumption, antibiotic susceptibility, DNase production, and hemolytic activity were evaluated.

#### 2.6.1. Antibiotic Susceptibility of LAB Strains

Antibiotic susceptibility was assessed using the standardized disk-diffusion method proposed by the Antibiogram Committee of the French Society of Microbiology [33] on MRS medium [34]. Seven antibiotics were tested: clindamycin (DA2, 2 µg/disk), erythromycin (E15, 15 µg/disk), doxycycline (DO30, 30 µg/disk), chloramphenicol (C30, 30 µg/disk), ampicillin (AMP10, 10 µg/disk), gentamicin (CN10, 10 µg/disk), and tetracycline (TE30, 30 µg/disk). After incubation, inhibition-zone diameters were measured and compared with breakpoint standards (Table S4) to determine susceptibility profiles.

#### 2.6.2. DNase and Hemolytic Activities

The ability of the strains to hydrolyze DNA was assessed using a well-diffusion method on DNase agar (Humeau, France). Hemolytic activity was assessed using a spot method on Columbia agar supplemented with sheep blood (Merck, Germany) [35]. A clear halo around a well or colony indicated a positive reaction. *Bacillus cereus* B8W8 was used as the positive control.

### 2.7. In Vivo Assessment of Plant Growth Promotion in Canola

The 32 strains were grown in MRS broth at 37 °C with shaking at 150 rpm. After 48 h, cells were harvested by centrifugation at 5000× *g* for 20 min and resuspended in phosphate-buffered saline (PBS). Three cell suspensions were prepared for each strain: 2.0 × 10^7^, 1.0 × 10^8^, and 1.0 × 10^9^ CFU mL^−1^. Uniform canola seeds were surface-sterilized by immersion in 95% ethanol for 30 s and washed five times with sterile distilled water. The seeds were dried in a laminar-flow hood and then soaked for 1 h in each bacterial suspension with shaking at 150 rpm. Control seeds were soaked in PBS.

A 1:1 (*v*/*v*) mixture of soil and sand was sterilized at 121 °C for 2 h. Disinfected pots were filled with the mixture, and one seed was sown per pot, with five replicate pots per treatment. The experiment followed a completely randomized design under greenhouse conditions (25 °C and a 16 h photoperiod). All successfully germinated plants from each treatment were gently harvested for analysis. Because not all of the five initially sown seeds germinated, the number of plants analyzed ranged from three to five per treatment. Then agronomic parameters, including plant total length, shoot and root length, were measured using a caliper, while shoot and root fresh and dry weights were measured using a balance [24].

### 2.8. Statistical Analysis

All quantitative data are expressed as the mean ± standard deviation (SD) of three independent replicates. Statistical analyses were performed using SPSS ( version 27.0 IBM Corp., NY, USA). Data normality was assessed using the Shapiro–Wilk test. Differences among LAB strains were analyzed by one-way analysis of variance (ANOVA), followed by Tukey’s honestly significant difference (HSD) post hoc test at *p* < 0.05.

To explore relationships among plant growth parameters and bacterial treatments, principal component analysis (PCA) was performed in R version 4.6.0. Variables were standardized before PCA with the prcomp() function in the stats package, and PCA biplots were generated using the factoextra package.

The plant growth promotion index (PGPI) was calculated following the integrative approach described by Agaras et al. (2015) [36], with modifications. Each growth parameter was standardized using a Z-score prior to calculation of the composite index:

Z*_ij_*= *x_ij_* − μ*_j_*/σ*_j_*

where *x_ij_* stands for the mean value of the growth parameter *j* of the strain *i*, while μ*_j_* represents the mean value of that parameter across the 32 strains and σ*_j_* is the corresponding standard deviation.

The PGPI index was then calculated using the following formula, and strains were ranked according to their PGPI values within each concentration:

*PGPI* = [*Z* (*RL*) + *Z* (*SL*) + *Z* (*TL*) + *Z* (*LN*) + *Z* (*RFW*) + *Z* (*SFW*) + *Z* (*RDW*) + *Z* (*SDW*)]/8 [36].

Where RL is root length; SL, shoot length; TL, total length; LN, leaf number; RFW, root fresh weight; SFW, shoot fresh weight; RDW, root dry weight; and SDW, shoot dry weight.

A heatmap of standardized traits and dose–response profiles was generated to visualize the contribution of individual growth parameters and the concentration-dependent behavior of selected strains. Data processing, Z-score standardization, PGPI calculation, ranking, and visualization were performed using Python version 3.13.5 (Python Software Foundation, Wilmington, DE, USA). Excel files were processed with pandas, numerical calculations were performed with NumPy version 2.0.0, and Matplotlib version 3.10.0 was used to generate PGPI ranking bar plots, the Z-score heatmap, and the dose–response line plot.

## 3. Results

### 3.1. In Vitro Assessment of LAB Antagonistic Activity Against Fungal Pathogens

**Figure 1:**
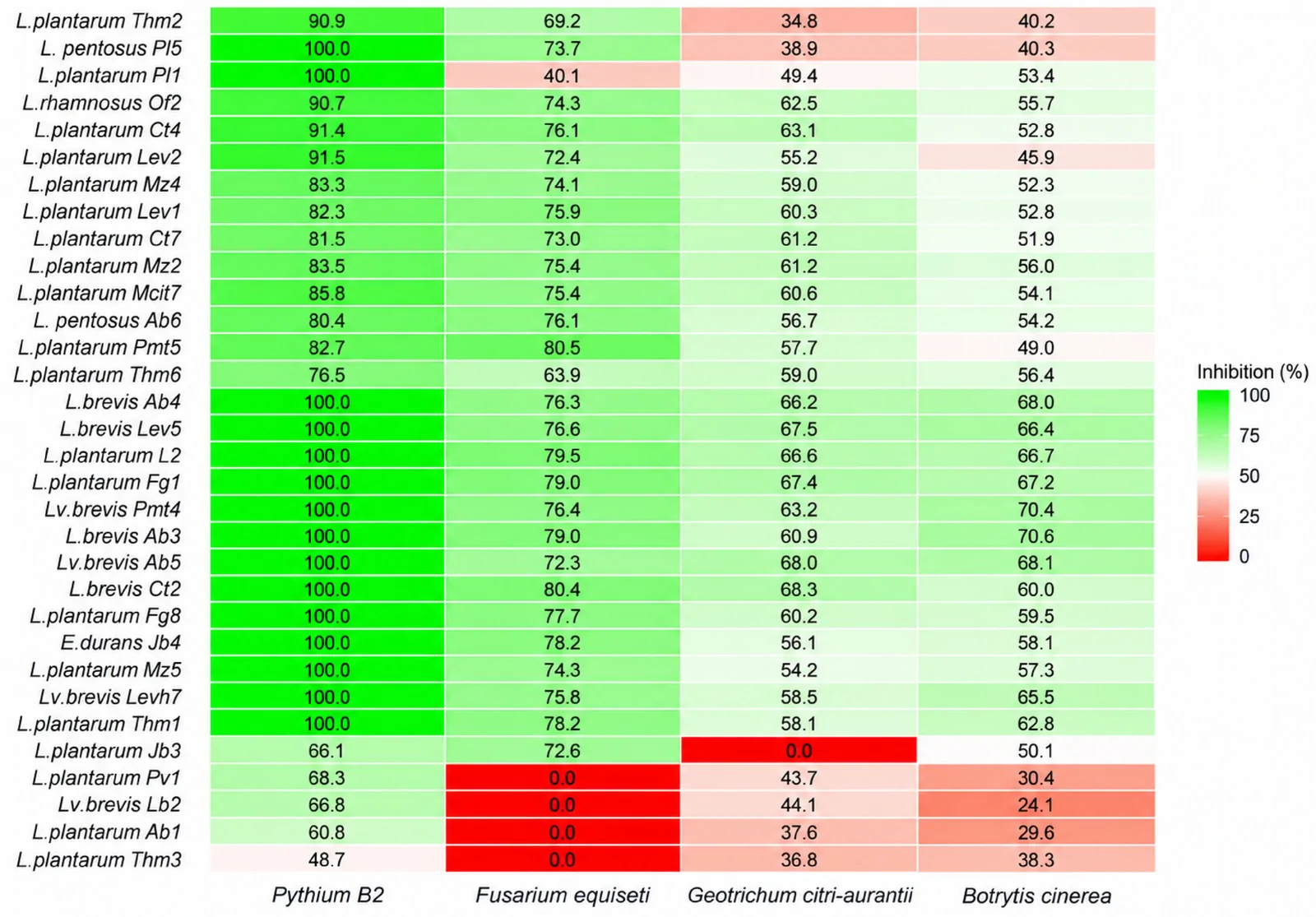
Heatmap showing the antagonistic activity of 32 LAB strains against the tested pathogens, expressed as percentage inhibition.

A total of 32 LAB isolates were tested against four major fungal and oomycete pathogens using a dual-culture assay. Inhibition rates were calculated when the control mycelium reached the edge of the plate, and the results are summarized in Figure 1. Inhibition varied significantly among strains and pathogens, ranging from no detectable activity to complete suppression of mycelial growth. The greatest antagonistic activity was observed against *P. heterothallicum*, for which almost 50% of the strains achieved complete inhibition. Nearly all strains also reduced the mycelial growth of *G. citri-aurantii* and *B. cinerea*. Approximately 40% of the LAB strains exhibited broad-spectrum activity against all four pathogens, with inhibition rates exceeding 60%, and overall inhibitory activity against *F. equiseti* remained relatively high.

### 3.2. Effect of pH Neutralization on Antimicrobial Activity

After the pH neutralization of the PDA medium, the LAB strains showed a complete loss of antifungal activity against all tested pathogens; no visible growth suppression of the studied pathogens was observed as all plates were covered with mycelium at the end of the experiment, whereas inhibitory effects had been detected under non-buffered conditions, as shown earlier, indicating that the LAB antifungal activity was primarily dependent on medium acidification and acidic metabolites production.

### 3.3. Assessment of In Vitro PGP Traits

Following the antagonism assays, the LAB strains were screened for *in vitro* PGP traits (Table 1). Significant differences among strains were detected for several quantitative traits. Phosphate solubilization indices ranged from 2.05 to 4.85. Strain Ab3 showed the highest value (4.85 ± 0.33), followed by Ct4 (4.53 ± 0.72), Of2 (4.36 ± 0.46), Ab6 (4.29 ± 0.22), and Mz2 (4.28 ± 0.24). These strains showed greater phosphate-solubilizing activity than Thm1, Ct2, and Thm2, whereas Pl5, Ab5, and Thm3 showed no detectable activity.

Cellulase activity also differed significantly among strains, with indices ranging from 3.24 to 6.92. Several strains had indices greater than 6.0; Lev5 showed the highest value (6.92 ± 0.08), followed by Pl5 and Of2 (6.32 ± 0.37 and 6.32 ± 0.07, respectively). In contrast, L2, Lev1, and Mcit7 showed significantly lower activity. Ammonia production was detected in fewer strains and ranged from 2.25 to 27.88 mg L^−1^. Pv1 produced the highest concentration (27.88 ± 0.02 mg L^−1^), followed by Ct4 (26.03 ± 0.04 mg L^−1^), Ab1 (25.75 ± 0.02 mg L^−1^), Pl1 (24.60 ± 0.04 mg L^−1^), and Lev2 (24.59 ± 0.02 mg L^−1^), whereas Ab4 produced the lowest concentration.

Acid phosphatase activity was detected in all strains, with no significant differences among strains (*p* > 0.05); values ranged from 0.17 to 0.32 µmol h^−1^. Alkaline phosphatase activity was not detected under the experimental conditions. Fewer than half of the strains produced hydrogen cyanide, with intensities ranging from weak (+) to moderate (++); strong production (+++) was recorded only for Ab3. Siderophore production was detected in only three strains, namely Ab3, Thm3, and Mcit7, with Ab3 showing the highest production level.

Protease, pectinase, chitinase, nitrogen fixation, and IAA production were not detected in any strain under the tested conditions. These findings demonstrate considerable functional variation among the LAB strains and identify isolates with strong plant-growth-promoting potential based on multifunctional biochemical profiles. Ab3 was among the most promising strains, combining the highest phosphate-solubilizing capacity with strong HCN production and substantial siderophore production.

Similarly, Ct4 combined high phosphate-solubilizing activity with elevated ammonia production, whereas Of2 combined strong phosphate solubilization and cellulase activity. Thm3 was distinguished by substantial siderophore production (53.38%), and Pv1 showed the greatest ammonia production. The coexistence of multiple PGP traits in these strains suggests that they may promote plant growth through complementary mechanisms, including enhanced nutrient availability, nutrient mobilization, and improved competitiveness.

### 3.4. PCR Detection of Lipopeptide- and Bacteriocin-Associated Genes

**Figure 2:**
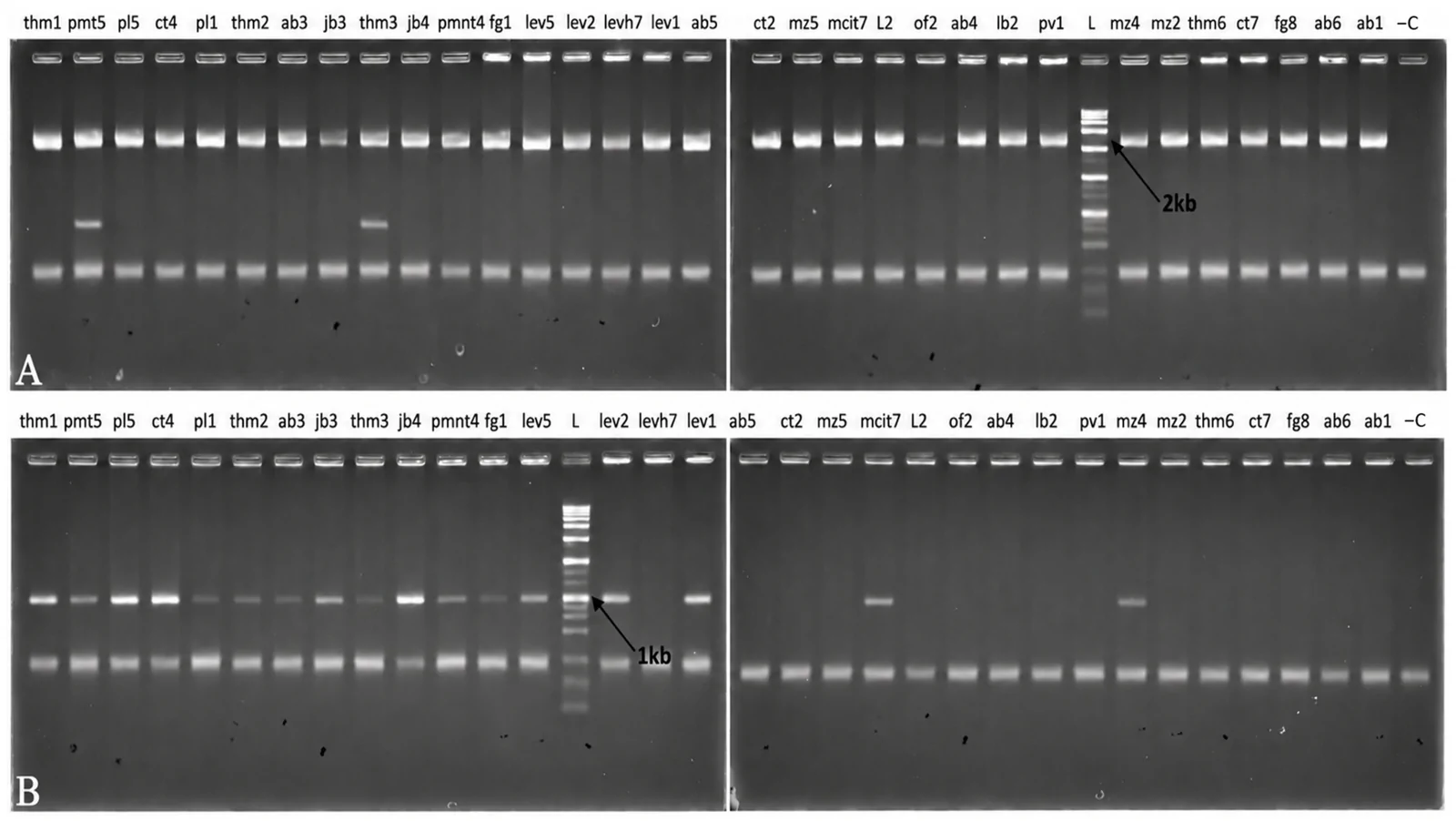
PCR-based detection of antimicrobial biosynthetic markers in 32 LAB strains. (**A**) Amplification profile of the iturin-associated gene using primers ITUP1F/ITUP2R. (**B**) Amplification profile of the subtilosin-associated gene using primers oSboP1N/oSboP2N. The expected amplicon sizes were approximately 2 kb for iturin and 1220 bp for subtilosin. L indicates the DNA ladder, and −C indicates the negative control.

Following electrophoretic separation and visualization, PCR screening of antimicrobial biosynthetic genes revealed distinct amplification patterns among the LAB strains (Figure 2).

For the iturin-associated gene, clear bands near the expected size of approximately 2 kb were observed in all strains except Of2, suggesting the presence of genes associated with iturin-like lipopeptide biosynthesis. The subtilosin A-associated gene had a more restricted distribution, with bands of approximately 1220 bp detected in only a subset of strains. No amplification was detected for the other screened genes. The absence of bands in the −C lanes indicated no detectable contamination during amplification. Overall, these results suggest that iturin and subtilosin may contribute to LAB antimicrobial activity, whereas the other screened antimicrobial biosynthetic genes were not detected.

### 3.5. Safety Assessment of LAB Strains

#### 3.5.1. Antibiotic Susceptibility of LAB Strains

The antibiotic susceptibility profiles of the LAB strains are summarized in Table 2. All strains were resistant to gentamicin (CN10), whereas most were susceptible to erythromycin (E15), chloramphenicol (C30), and ampicillin (AMP10), with inhibition-zone diameters generally exceeding 25 mm. Responses to clindamycin (DA2), doxycycline (DO30), and tetracycline (TE30) were more variable among strains. Most strains were resistant to clindamycin, whereas susceptibility to doxycycline predominated, although some resistant phenotypes were also observed. Responses to tetracycline were predominantly intermediate, with several strains classified as susceptible. No strain was resistant to all tested antibiotics. Overall the susceptibility profiles were heterogeneous, indicating that antibiotic resistance should be considered on a strain-specific level rather than a uniform characteristic of the LAB collection. The observed resistant and intermediate phenotypes warrant cautious interpretation as the phenotypic susceptibility testing alone does not distinguish intrinsic resistance from acquired resistance mechanisms.

#### 3.5.2. DNase and Hemolytic Activities

**Figure 3:**
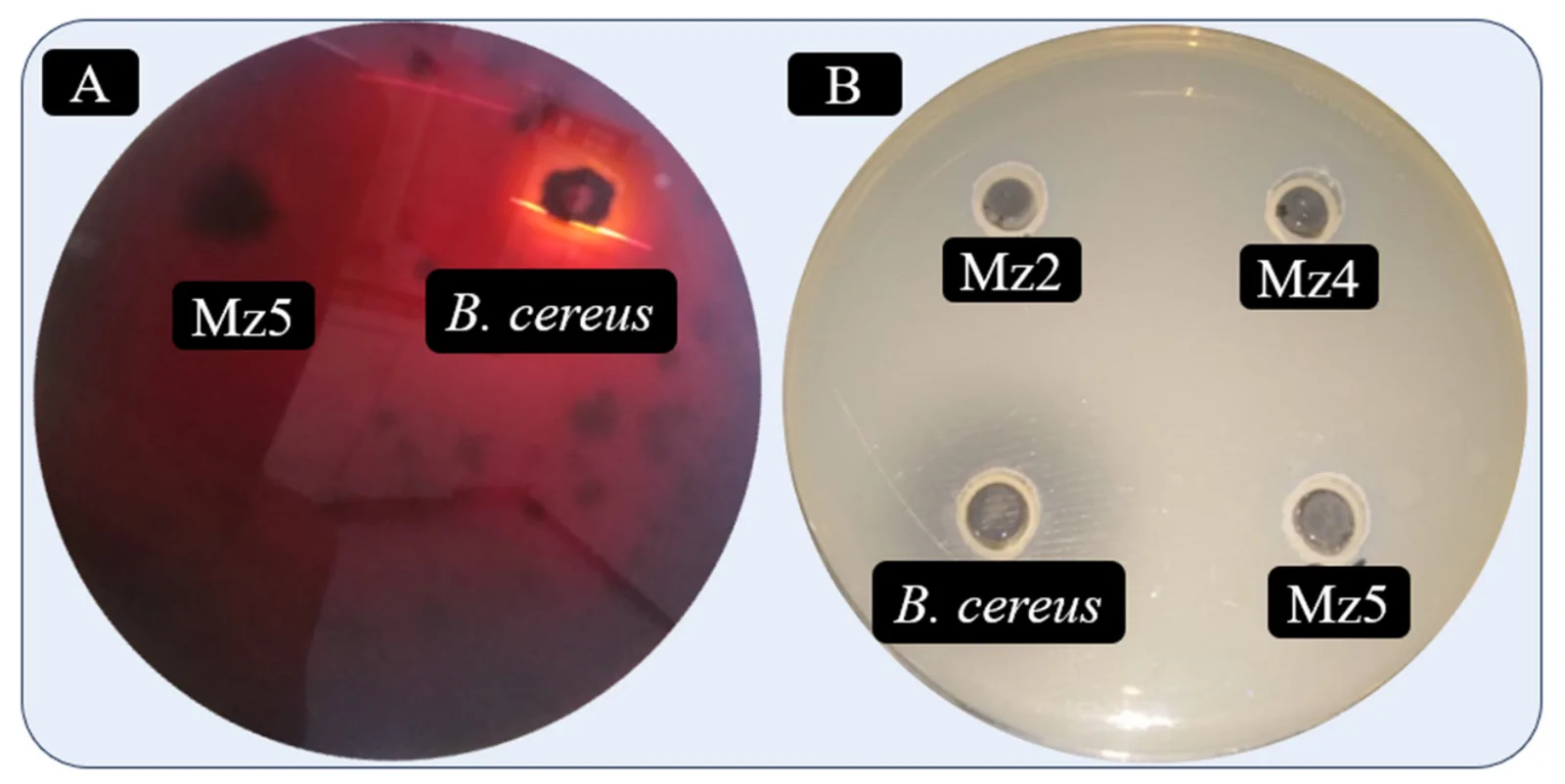
(**A**) Strain Mz5 showing no detectable hemolytic activity compared with the positive control, *B. cereus*. (**B**) Strains Mz2, Mz4, and Mz5 showing no detectable DNase activity compared with the positive control.

After incubation at 37 °C for 48 h, clear halos around colonies were observed only for the positive control, *B. cereus* (Figure 3). None of the 32 LAB strains produced clear zones around inoculation spots or agar wells, indicating no detectable hemolytic or DNase activity.

### 3.6. In Vivo Assessment of Plant Growth Promotion in Canola

To further evaluate the LAB strains, a greenhouse experiment was conducted to assess *in vivo* plant growth promotion in canola inoculated with three concentrations of each strain. Principal component analysis (PCA) was used to examine relationships among the measured parameters and the overall variation in plant growth responses.

**Figure 4:**
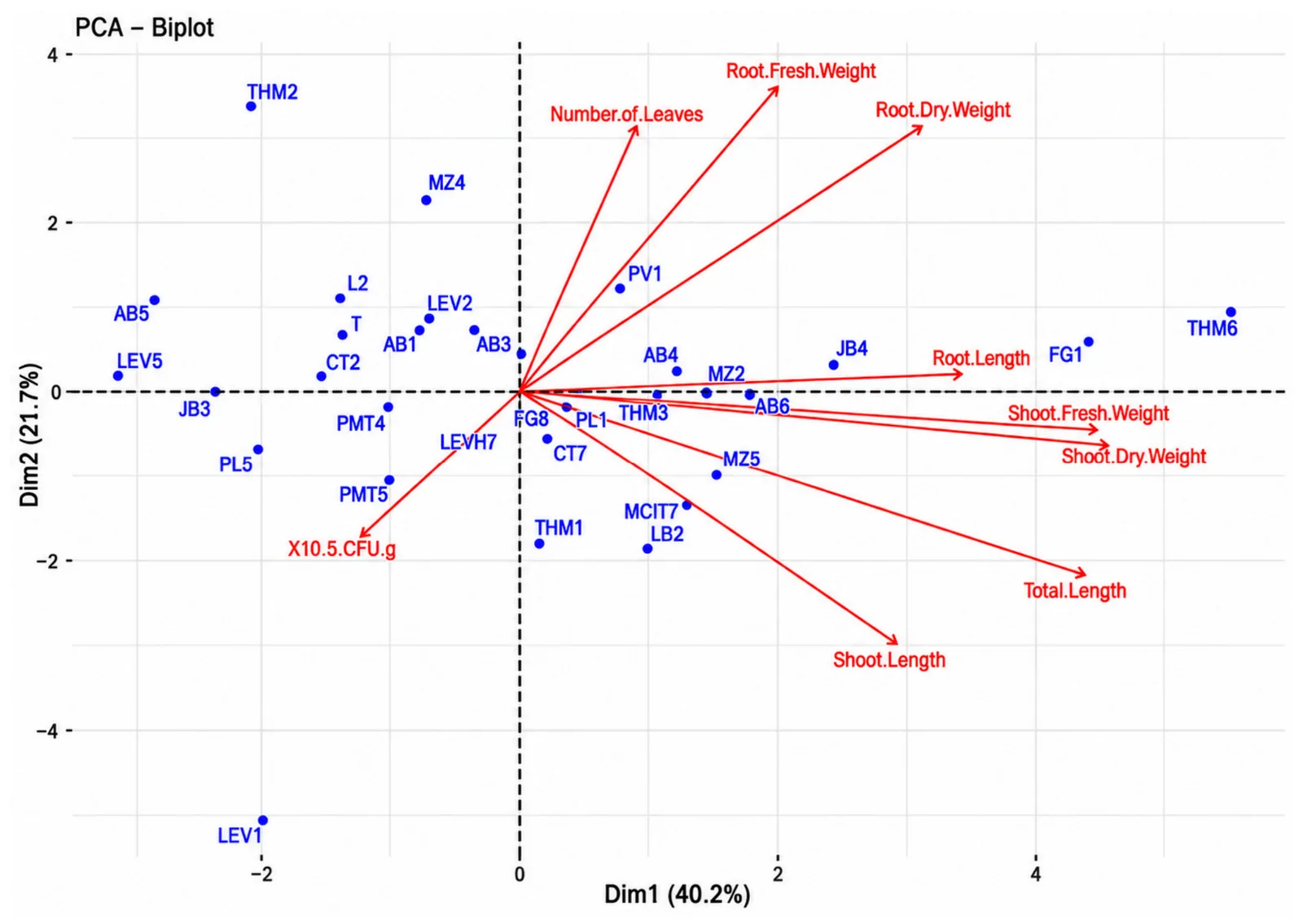
PCA biplot showing the effects of LAB inoculation at 2.0 × 10^7^ CFU mL^−1^ on root and shoot development in *B. napus*. PC1 and PC2 explained 40.2% and 21.7% of the total variance, respectively. Arrows represent morphological variables, and points represent bacterial strains.

At 2 × 10^7^ CFU mL^−1^, the first two principal components explained 61.9% of the total variance: Dim1 accounted for 40.2%, and Dim2 accounted for 21.7% (Figure 4). Shoot-related variables and total plant length loaded positively on Dim1. Root length also contributed positively, although its vector was closer to the origin. Dim2 was mainly associated with root biomass parameters and leaf number, which loaded positively and projected into the upper quadrants.

The strains varied considerably in their associations with plant growth parameters. Thm6 and Fg1 were positioned near shoot-related variables on the positive side of Dim1, whereas Pv1 and Ab4 were closer to root-associated variables and leaf number in the upper-right quadrant. Pl5, Jb3, and Pmt5 were located on the negative side of Dim1, indicating a different association pattern. Of2, Ct7, and Thm3 were closer to the origin, suggesting weaker contributions to the variation explained by the first two components.

**Figure 5:**
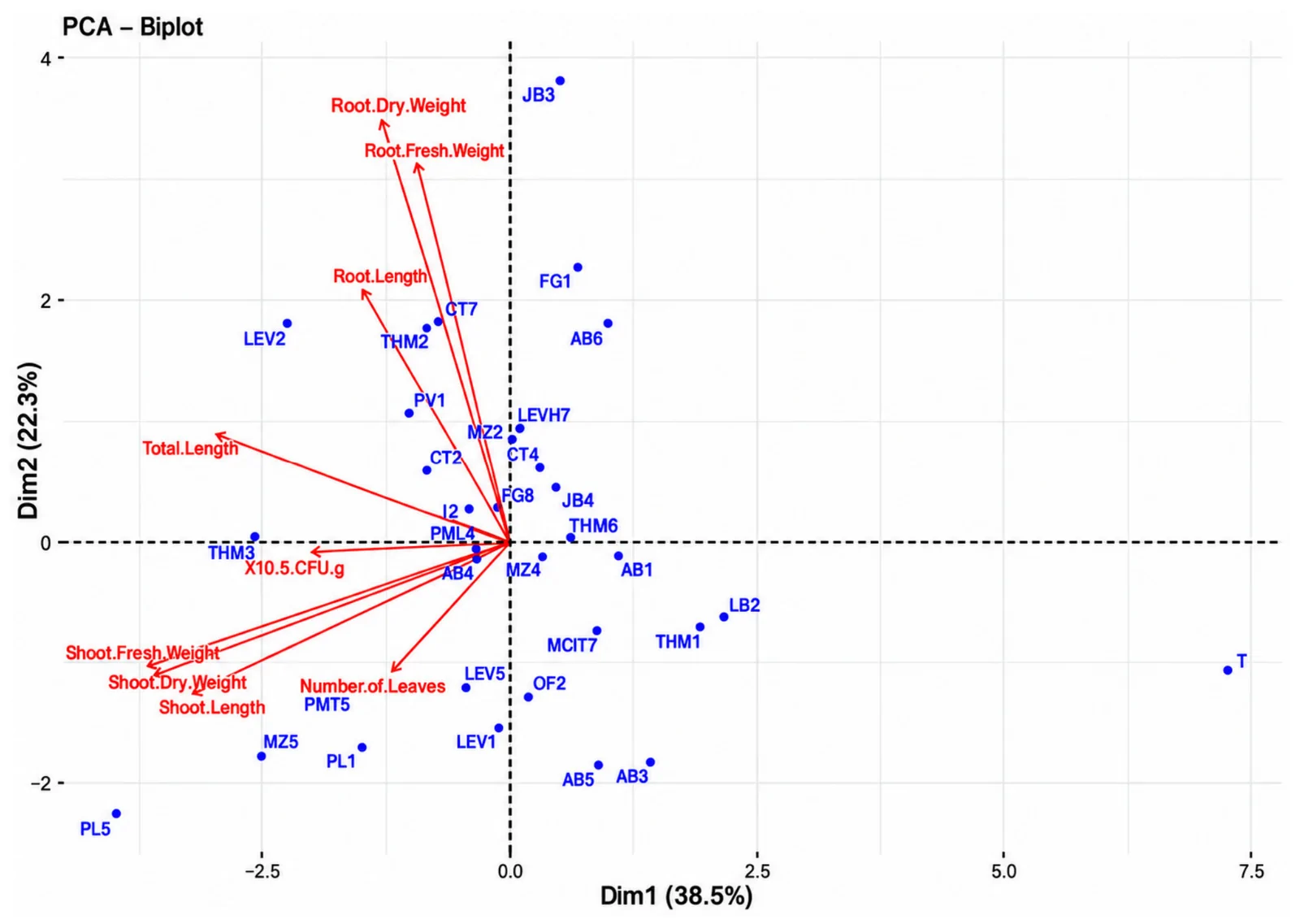
PCA biplot showing the effects of LAB inoculation at 1 × 10^8^ CFU mL^−1^ on root and shoot development in *B. napus*. PC1 and PC2 explained 38.5% and 22.3% of the total variance, respectively. Arrows represent morphological variables, and points represent bacterial strains.

At 1 × 10^8^ CFU mL^−1^, the first two principal components explained 60.8% of the total variance: Dim1 accounted for 38.5%, and Dim2 accounted for 22.3% (Figure 5). The distribution of variables showed a clear organization of plant growth parameters. Root biomass parameters loaded positively on Dim2 and clustered in the upper-left quadrant. In contrast, shoot-related variables, including leaf number, were oriented toward the lower-left quadrant, indicating a distribution distinct from that of the root-related parameters.

Total plant length was clearly separated from the other plant parameters on the negative side of Dim1, indicating a distinct contribution. The bacterial strains were distributed heterogeneously across the biplot. JB3 and FG1 were located in the upper quadrants near root-related variables, whereas PL5, PMT5, and MZ5 were positioned in the lower quadrants near shoot-related parameters. CT4, LEVH7, and MZ2 were close to the origin, suggesting limited contributions to the variation explained by the first two components. These results suggest that, at this inoculum concentration, some strains were more strongly associated with root growth and others with shoot development.

**Figure 6:**
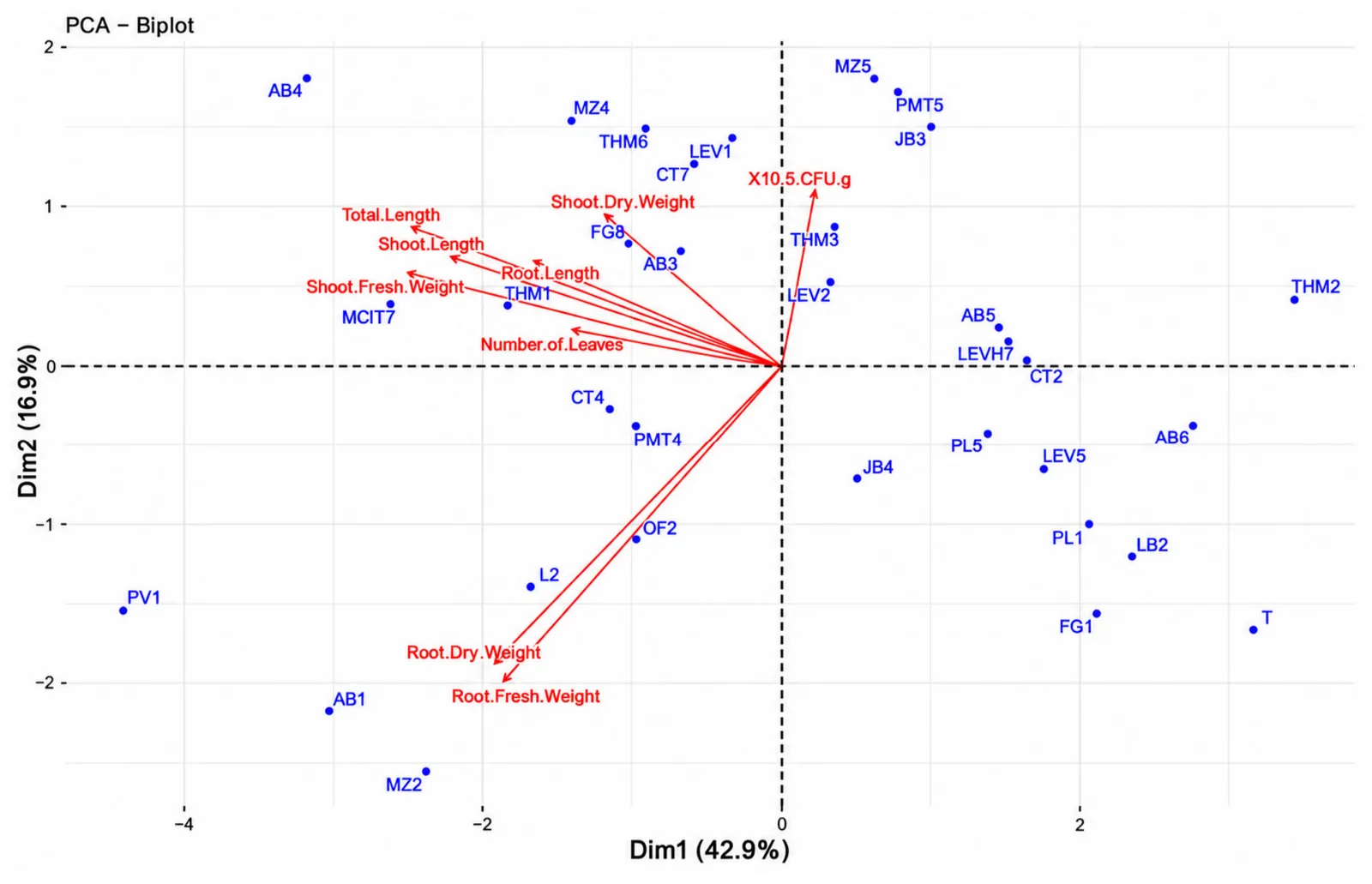
PCA biplot showing the effects of LAB inoculation at 1 × 10^9^ CFU mL^−1^ on root and shoot development in B. napus. PC1 and PC2 explained 42.9% and 16.9% of the total variance, respectively. Arrows represent morphological variables, and points represent bacterial strains.

At 1 × 10^9^ CFU mL^−1^, the first two principal components explained 59.8% of the total variance: Dim1 accounted for 42.9%, and Dim2 accounted for 16.9% (Figure 6).

The projection of variables showed a clear organization of plant growth parameters. Most shoot-related parameters, including total plant length and leaf number, clustered on the negative side of Dim1. Root length had a similar orientation, whereas the root biomass variables were clearly separated and projected onto the negative side of Dim2, indicating a distinct distribution relative to the other growth parameters.

The bacterial strains were widely dispersed across the biplot. MZ5, JB3, and PMT5 were positioned in the upper-right quadrant, whereas PV1, MZ2, and AB1 were located near the root biomass variables. CT4, OF2, and PMT4 were closer to the origin, suggesting limited contributions to the variation explained by the first two components.

At the highest concentration, root biomass variables were clearly separated from shoot-related variables, and the bacterial strains were widely dispersed across the biplot.

To complement the PCA and integrate the overall growth response to LAB inoculation, the plant growth promotion index (PGPI) was calculated for each strain at the three different concentrations.

**Figure 7:**
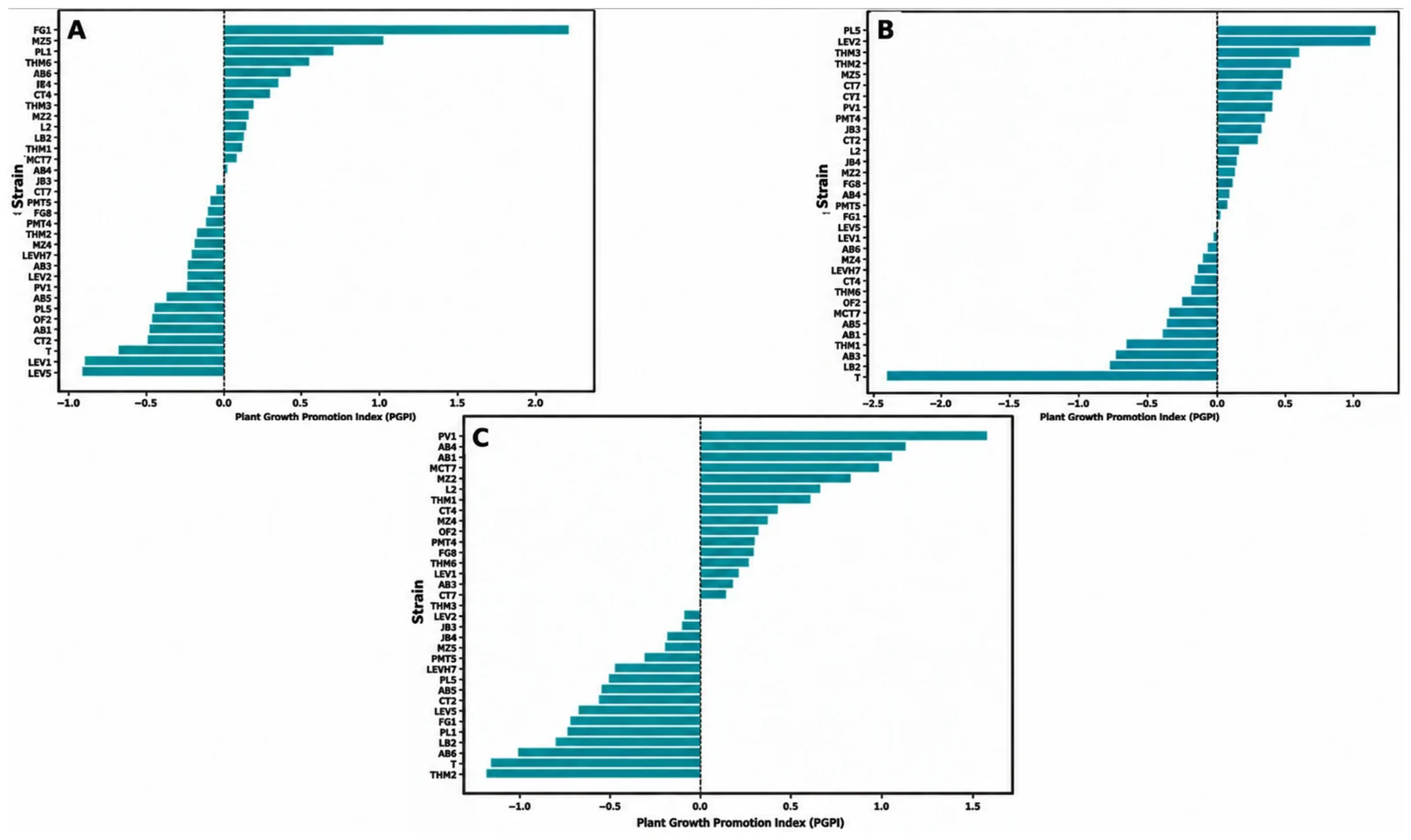
PGPI rankings of 32 LAB strains at each concentration: (**A**) 2.0 × 10^7^ CFU mL^−1^, (**B**) 1.0 × 10^8^ CFU mL^−1^, and (**C**) 1.0 × 10^9^ CFU mL^−1^. Positive PGPI values indicate above-average overall plant growth promotion, whereas negative values indicate below-average effects.

The PGPI rankings showed marked strain- and concentration-dependent patterns. At 2× 10^7^ CFU mL^−1^ (Figure 7A), several strains, including Mz5, Pl1, Thm6, and Ab6, had positive PGPI values, indicating growth-promoting effects at a low bacterial concentration. Fg1 showed the strongest overall growth-promoting effect at this concentration.

At 1× 10^8^ CFU mL^−1^ (Figure 7B), strain rankings shifted markedly, and Lev2 and Pl5 emerged as the best-performing strains. This pattern indicates that some strains required a higher cell density to achieve their optimal growth-promoting potential. More strains had positive PGPI values at this concentration than at the lowest concentration, although the magnitude of the response varied among strains.

At 1 × 10^9^ CFU mL^−1^ (Figure 7C), strain rankings shifted again. Several strains that performed well at lower concentrations showed markedly reduced effects at the highest concentration. FG1, Mz5, and PL5, which had positive PGPI values at lower concentrations, had negative values at 1.0 × 10^9^ CFU mL^−1^. In contrast, Pv1, Ab1, Ab4, Mcit7, and Mz2 maintained or improved their PGPI values, indicating stronger growth-promoting effects at the highest inoculum density.

Based on mean PGPI values across the three concentrations, the ten most promising strains were Pv1, Fg1, Mz5, Ab4, Mz2, L2, Lev2, Thm3, Mcit7, and Thm6. However, no strain consistently ranked first at all concentrations, further demonstrating the concentration-dependent performance of the LAB collection.

**Figure 8:**
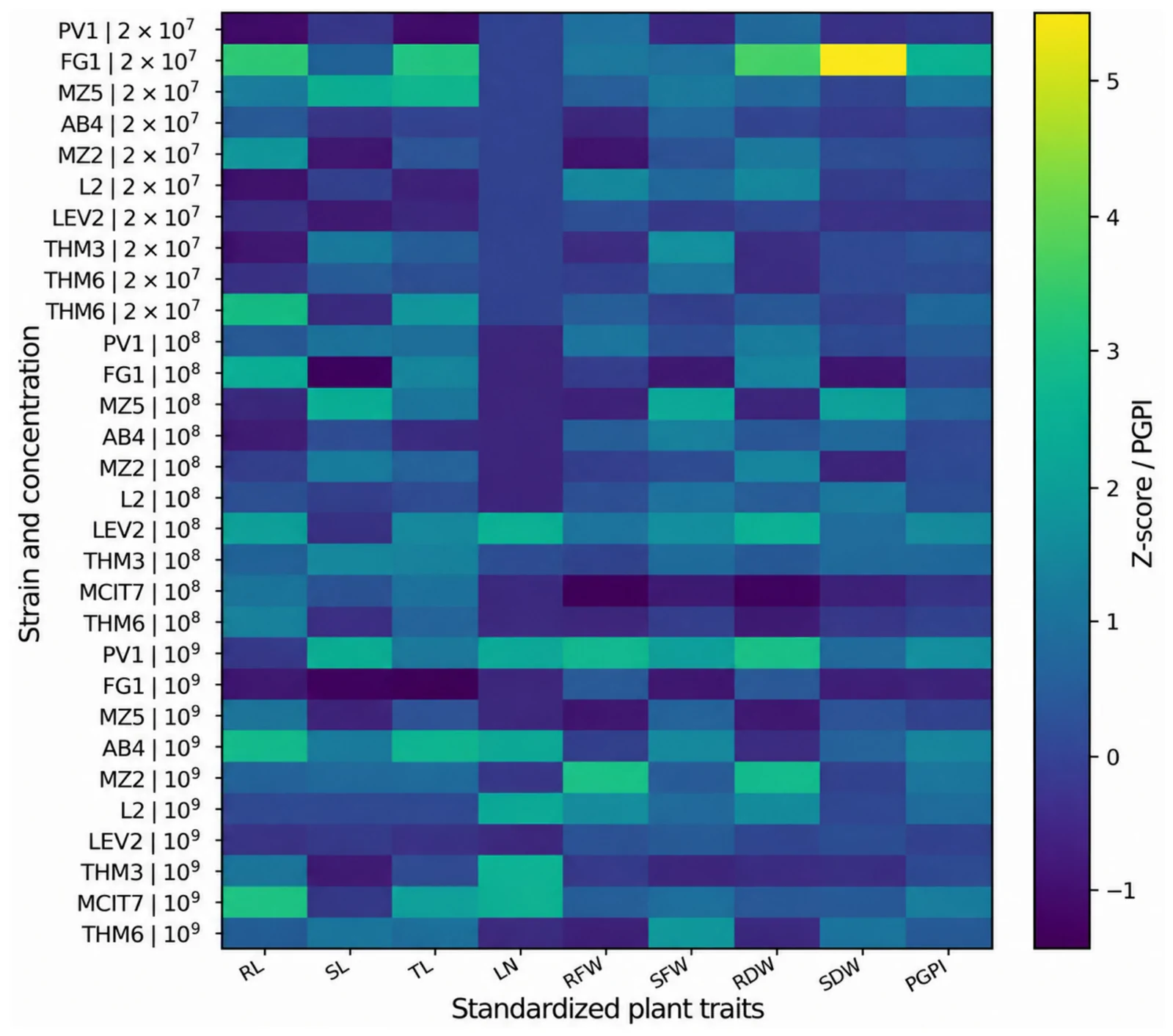
Z-score heatmap of individual plant growth parameters and overall PGPI. Positive values indicate an above-average response for the corresponding trait, whereas negative values indicate below-average performance.

The Z-score heatmap (Figure 8) further clarified the contribution of each plant trait to the overall PGPI. Strains with high PGPI values generally showed positive responses across multiple parameters, indicating balanced stimulation of shoot growth, root development, biomass accumulation, and leaf production. Some strains with lower PGPI values nevertheless showed strong positive responses for specific traits, suggesting more specialized effects on shoot or root development.

**Figure 9:**
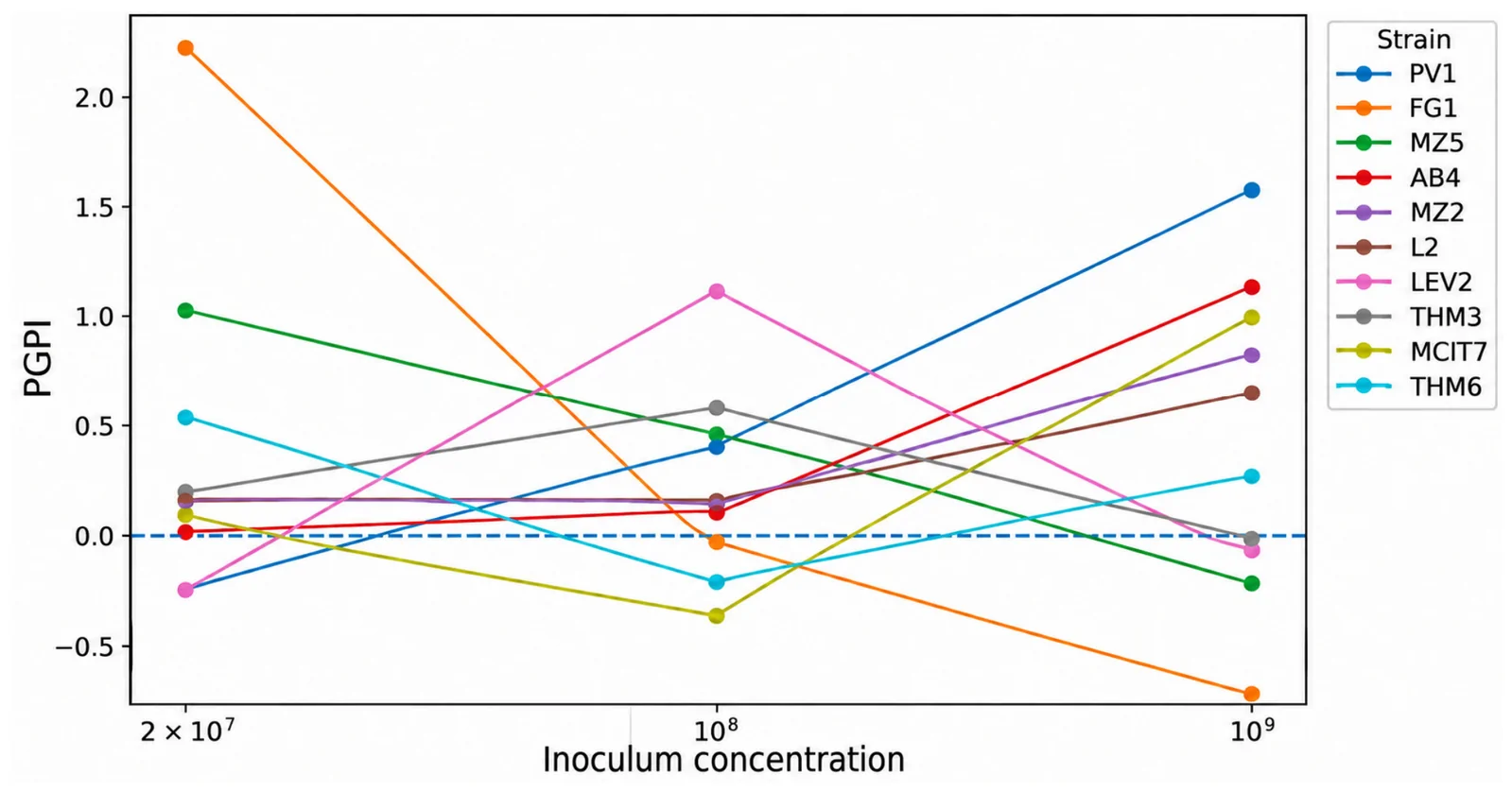
Dose–response profiles of ten selected LAB strains based on mean PGPI values across three inoculum concentrations. Lines show changes in overall plant-growth-promoting performance from 2× 10^7^ to 1× 10^9^ CFU mL^−1^.

The dose–response profiles (Figure 9) confirmed the strong concentration-dependent effects of LAB inoculation on *B. napus* growth. PGPI trajectories varied markedly among strains. FG1 and Mz5 performed best at the lowest concentration (2 × 10^7^ CFU/mL), and their PGPI values declined at higher concentrations. Thm6 and Mcit7 performed least well at 1.0 × 10^8^ CFU mL^−1^, whereas Lev2 and Thm3 performed best at this intermediate concentration. Pv1, Ab4, Mz2, and L2 showed stable or increasing PGPI values across the first two concentrations and performed best at 1 × 10^9^ CFU mL^−1^. These contrasting patterns confirm that LAB-mediated plant growth promotion did not follow a linear dose–response relationship but instead depended on a strain-specific optimal concentration. Dose optimization is therefore essential before candidate strains undergo further greenhouse and field validation.

## 4. Discussion

This study evaluated the potential of LAB as multifunctional bioinoculants by integrating antifungal activity, plant-growth-promoting biochemical traits, molecular screening of antimicrobial markers, safety assessment, and greenhouse evaluation of plant-growth-promoting effects using *Brassica napus* as a model plant. The effects were strongly strain- and concentration-dependent, indicating that bioinoculant performance cannot be generalized at the species level and must instead be evaluated for each strain and application dose. The selection of LAB strains that combined plant growth promotion effects with *in vitro* antifungal activity supports their further development as dual-function bioinoculants for sustainable crop production.

Within this integrated framework, the dual culture assays provided an initial measure of the biocontrol potential of the strains. Their inhibition patterns against the four pathogens revealed both disease-suppressive potential and functional specificity.

The strong antifungal activity observed in this study highlights the potential of several strains for further investigation as candidate biocontrol agents. However, inhibition varied among strains and pathogens, supporting the interpretation that LAB antagonism is strain-specific rather than a universal property of this bacterial group. This finding is consistent with previous reports of wide variation in the inhibitory spectra of LAB species and strains according to their origin, metabolic profile, and target pathogen [37,38].

Among the four pathogens, *Pythium heterothallicum* was the most sensitive, whereas inhibition of the other pathogens was more variable. Similar strain- and pathogen-dependent patterns have been reported previously. For example, Kharazian et al. (2017) described varying levels of inhibition of several phytopathogenic fungi, including *Penicillium* spp., *Fusarium verticillioides*, *Pythium aphanidermatum*, and *Verticillium dahliae*, by *Lactobacillus* strains isolated from corn silage [39].

The antifungal activity of these strains appeared to be driven primarily by acidification, as indicated by the complete loss of activity after the medium was neutralized. This interpretation is consistent with the established role of LAB-derived organic acids, particularly lactic and acetic acids, which lower extracellular pH, disrupt the proton motive force and fungal membrane integrity, and impair intracellular pH homeostasis [38,39].

Other studies have reported LAB-mediated inhibition of citrus fungal pathogens, including *Geotrichum citri-aurantii*, through the production of organic acids, particularly lactic, acetic, and phenyllactic acids, with lactic acid showing greater antifungal efficacy than the other acids [40]. Similar organic acid-mediated activity has been reported for *Lactiplantibacillus plantarum* and other LAB species that produce hydroxyphenyllactic acid, phenyllactic acid, and cyclic dipeptides active against *Penicillium*, *Aspergillus*, and *Fusarium* species [41,42,43].

The marked variation in inhibition profiles among isolates and target pathogens suggests that antifungal activity results from mechanisms extending beyond organic acid production. The detection of putative markers associated with iturin and subtilosin A biosynthesis suggests the possible involvement of additional antimicrobial metabolites. LAB can produce antifungal peptides, bacteriocin-like compounds, fatty acids, volatile compounds, and other inhibitory molecules and can also suppress fungal growth through competition for nutrients and space [37,38,44]. Antagonism by the active strains may therefore result from synergistic mechanisms in which acidification is a major driver and strain-specific metabolites or competitive interactions determine the breadth and intensity of inhibition.

Although the antifungal profiles demonstrate the capacity of selected LAB strains to inhibit mycelial growth of the tested pathogens *in vitro*, their value as dual-function bioinoculants also depends on their capacity to support plant vigor through complementary nutritional and ecological mechanisms. Screening of *in vitro* PGP traits revealed the multifunctional nature of several strains and strengthened their relevance as bioinoculant candidates.

Several strains combined complementary mechanisms involved in organic matter transformation, nutrient mobilization, and indirect pathogen suppression. Ab3 combined the highest phosphate-solubilizing activity with high siderophore production and strong HCN production, whereas Ct4 combined high ammonia production with substantial phosphate solubilization. Of2 combined strong phosphate solubilization with cellulase activity, Thm3 produced substantial amounts of siderophores, and Pv1 showed the highest ammonia production. The coexistence of multiple traits is particularly relevant because plant growth promotion under field conditions rarely depends on a single mechanism and instead results from coordinated processes [9,10,45,46].

Phosphate solubilization and phosphatase activities detected in several LAB strains may increase plant access to poorly available mineral and organic phosphorus pools. Ammonia production may increase nitrogen availability, whereas siderophore production may improve iron acquisition by plants while restricting Fe^3+^ availability to pathogens. In addition, the high cellulase activity of most strains suggests potential roles in organic matter turnover and adaptation to root-associated niches [47,48,49].

Hydrogen cyanide (HCN) production may further contribute to the biocontrol potential of selected LAB strains, particularly Ab3, by inhibiting fungal respiration through interference with cytochrome c oxidase and other metalloenzymes, a mechanism commonly associated with bacterial cyanogenesis [50,51]. HCN may also influence nutrient availability, particularly phosphate mobilization through metal chelation [30]. The combination of these traits may improve plant ecological fitness and the functional stability of inoculants under variable field conditions [9,52,53]. Further *in vivo* validation is nevertheless required to confirm the biological relevance and expression of these traits under more complex conditions.

The absence of detectable nitrogen fixation, IAA production, alkaline phosphatase activity, and lytic enzyme production does not necessarily demonstrate the absence of the corresponding genetic or functional potential; it may instead reflect a lack of activity under the tested conditions [49,54]. These results help define the functional boundaries of the strains. The *in vitro* findings also provide a mechanistic basis for interpreting the greenhouse experiment, in which the biological relevance of these traits was evaluated through the overall growth response of *B. napus*.

PCA revealed functional differentiation among the LAB strains, as shown by the separation of shoot- and root-related variables in the biplots. This distribution suggests that LAB inoculation induced distinct developmental responses rather than uniform growth promotion: some strains were more strongly associated with shoot growth, whereas others were associated with root development. Such organ-specific responses are common in plant–microbe interactions, in which bacterial effects may depend on phytohormonal modulation, root colonization, nutrient mobilization, and plant physiological status [9,46,55].

The clustering of morphologically related traits indicates coordinated plant responses to LAB inoculation. The association of shoot length with shoot fresh and dry weights reflects coordinated aboveground growth, whereas the clustering of root fresh and dry weights indicates concerted stimulation of root biomass. Shoot and root development are physiologically interconnected through nutrient uptake, carbon allocation, and hormonal crosstalk. Different LAB strains may therefore promote growth through distinct mechanisms and produce different effects on belowground and aboveground development [49,55,56].

The broad dispersion of LAB strains in the PCA space reflects substantial variation in plant growth responses. Some strains were associated with shoot growth and others with root development, whereas strains near the origin had profiles closer to the multivariate average [46]. These differences were also evident in the Z-score heatmap of individual growth parameters (Figure 8), in which each selected strain showed a distinct trait-level signature. Strains with positive Z-scores across several traits may be considered balanced growth promoters and may be suitable for crops in which overall biomass is desired. Shoot-oriented strains may be particularly relevant to leafy vegetables, cereals, canola, and forage crops, in which aboveground biomass contributes directly to productivity [57,58]. Root-oriented strains may be more suitable for applications targeting belowground growth. For example, Mz2 at the highest concentration could be useful for root and storage crops such as beetroot, carrot, radish, turnip, or potato, consistent with studies showing that PGPR can improve root function, nutrient acquisition, and root yield [55,59]. These findings emphasize the need to select strains according to the intended agronomic application.

The PGPI ranking further demonstrated the nonlinear response of *B. napus* to LAB inoculation. Some strains, such as Fg1, showed their highest growth-promoting activity at the lowest inoculum concentration, whereas others performed best at an intermediate concentration (Lev2) or high inoculum density (Pv1). Nine of the ten best-performing isolates belonged to *Lactiplantibacillus plantarum*, yet their dose–response profiles and growth-promoting efficiencies differed markedly. This substantial intraspecific variation suggests that strain identity was more influential than species affiliation, consistent with reports that closely related PGPR isolates may differ substantially in metabolite production and root colonization. Strain-level selection is therefore more informative than species-level generalization [9,55].

The dose–response profiles (Figure 9) show that LAB-mediated plant growth promotion did not follow a linear relationship with inoculum concentration. Increasing inoculum density did not consistently improve plant performance; instead, each strain appeared to have an optimal concentration. Fg1 and Mz5 performed best at the lowest concentration; Pv1, Ab4, Mz2, and L2 maintained or improved their effects at the highest concentration; and Lev2 and Thm3 performed best at the intermediate concentration. This nonlinear behavior suggests that LAB–plant interactions depend on a balance among cell density, metabolite production, root colonization, and plant physiological tolerance. Similar dose-sensitive patterns have been reported for PGPR and attributed to root-niche saturation, excessive metabolite accumulation, nutrient competition, or attenuation of beneficial effects [9,52,60].

This pattern is not universal. Yavarian et al. (2021), for example, reported increased shoot length when PGP bacterial densities increased from 1× 10^6^ to 1× 10^7^ CFU/g soil [60]. Pagliarini et al. (2023) found that a higher dose of *Bacillus* sp. VWC18 substantially increased root fresh and dry weights in basil [61]. Our findings therefore indicate that dose–response relationships are strain-, crop-, and context-dependent rather than contradicting reports of dose-dependent stimulation.

Collectively, these findings support the potential of the investigated LAB strains as bioinoculant candidates within the broader group of plant-growth-promoting bacteria. In addition to conventional PGP and biocontrol traits, eligible LAB strains or species offer practical advantages, including a long history of use in food applications, GRAS or QPS status, and consumer acceptance [13,38,62]. The preliminary safety profile observed here supports this potential: none of the strains showed hemolytic or DNase activity, and no broadly concerning antibiotic resistance profiles were detected; however, these assays represent just a preliminary safety screening, and further characterization must be conducted, particularly for strains presenting resisting or intermediate phenotypes.

This study has several limitations. Although selected LAB strains showed significant *in vitro* antifungal activity, no *in planta* pathogen challenge experiment was performed. Therefore, further studies involving plant-pathogen challenge assays should be conducted. In addition, Greenhouse validation was conducted under controlled conditions using a single plant species, and rhizosphere colonization, persistence in nonsterile soil, antimicrobial gene expression, and metabolite production were not directly assessed. Future work should therefore include field-scale validation, analysis of root colonization dynamics, genome-based safety assessment, and metabolomic characterization of antifungal and plant-growth-promoting mechanisms. These investigations are needed to advance the most promising LAB strains from functional screening to reliable, safe, and application-oriented bioinoculants.

## 5. Conclusions

This study demonstrates that LAB are promising candidates for dual-function bioinoculants that combine *in vitro* antifungal activity, multifunctional plant-growth-promoting traits, *in vivo* growth promotion, and a favorable preliminary safety profile. Several strains showed broad *in vitro* antagonistic activity against phytopathogens and complementary traits potentially involved in nutrient mobilization and indirect pathogen suppression, including phosphate solubilization, acid phosphatase and cellulase activities, and ammonia, siderophore, and HCN production. *In vivo* validation using *B. napus* showed that growth promotion was highly strain- and concentration-dependent, with different strains associated with shoot development, root development, or balanced whole-plant growth. Plant growth promotion did not follow a linear dose–response relationship, indicating that strain selection should be accompanied by optimization of inoculum concentration before field evaluation. The selected LAB strains therefore warrant further development as multifunctional bioinoculants, although their performance must be validated in nonsterile soil and under field conditions. Their colonization dynamics and metabolite-mediated mechanisms also require further investigation.

## Figures and Tables

**Table 1:** Summary of the *in vitro* plant-growth-promoting traits of the tested LAB strains.

LAB Strain	Phosphate Solubilization Index	Acid Phosphatase (µmol h^−1^)	Alkaline Phosphatase (µmol h^−1^)	Cellulase Activity Index	Ammonia Production (mg L^−1^)	HCN Production	Siderophore Production (%)	Amylase Activity Index	Pectinase Activity Index	Protease Activity Index	Nitrogen Fixation	Chitinase Activity Index	IAA Production
Levh7	3.10 ± 0.17 ^c–f^	0.31 ± 0.02 ^a^	-	5.63 ± 0.12 ^j–l^	16.80 ± 0.03 ^i^	-	- ^d^	-	-	-	-	-	-
Pl1	2.96 ± 0.11 ^cde^	0.27 ± 0.01 ^a^	-	6.03 ± 0.06 ^i–l^	24.60 ± 0.04 ^r^	-	- ^d^	-	-	-	-	-	-
Pl5	- ^a^	0.21 ± 0.01 ^a^	-	6.32 ± 0.37 ^lm^	- ^a^	++	- ^d^	-	-	-	-	-	-
JB3	3.49 ± 0.20 ^e–i^	0.25 ± 0.05 ^a^	-	5.31 ± 0.27 ^e–i^	- ^a^	-	- ^d^	-	-	-	-	-	-
JB4	4.00 ± 0.04 ^h–l^	0.24 ± 0.01 ^a^	-	5.25 ± 0.27 ^e–h^	- ^a^	++	- ^d^	-	-	-	-	-	-
L2	4.02 ± 0.06 ^h–l^	0.25 ± 0.02 ^a^	-	3.24 ± 0.22 ^b^	13.41 ± 0.03 ^e^	++	- ^d^	-	-	-	-	-	-
LB2	3.90 ± 0.15 ^h–l^	0.32 ± 0.01 ^a^	-	5.30 ± 0.05 ^e–h^	21.16 ± 0.02 ^n^	-	- ^d^	-	-	-	-	-	-
Lev1	3.54 ± 0.08 ^d–i^	0.31 ± 0.05 ^a^	-	3.54 ± 0.30 ^bc^	- ^a^	-	- ^d^	-	-	-	-	-	-
Lev2	4.11 ± 0.06 ^i–l^	0.23 ± 0.00 ^a^	-	5.20 ± 0.13 ^e–h^	24.59 ± 0.02 ^r^	-	- ^d^	-	-	-	-	-	-
Lev5	3.78 ± 0.06 ^g–k^	0.29 ± 0.03 ^a^	-	6.92 ± 0.08 ^m^	16.68 ± 0.02 ^h^	-	- ^d^	-	-	-	-	-	-
Pmt4	2.79 ± 0.20 ^c^	0.30 ± 0.05 ^a^	-	6.05 ± 0.22 ^j–l^	8.26 ± 0.02 ^c^	++	- ^d^	-	-	-	-	-	-
Pmt5	3.00 ± 0.35 ^c–f^	0.27 ± 0.02 ^a^	-	5.86 ± 0.16 ^h–l^	- ^a^	+	- ^d^	-	-	-	-	-	-
Fg1	3.43 ± 0.19 ^d–h^	0.28 ± 0.08 ^a^	-	4.87 ± 0.41 ^def^	14.30 ± 0.01 ^f^	-	- ^d^	-	-	-	-	-	-
Fg8	3.15 ± 0.08 ^c–g^	0.26 ± 0.04 ^a^	-	4.76 ± 0.34 ^de^	23.06 ± 0.01 ^q^	+	- ^d^	-	-	-	-	-	-
Thm1	2.60 ± 0.04 ^bc^	0.23 ± 0.00 ^a^	-	6.08 ± 0.07 ^j–l^	20.38 ± 0.02 ^l^	+	- ^d^	-	-	-	-	-	-
Thm2	2.05 ± 0.06 ^b^	0.17 ± 0.04 ^a^	-	- ^a^	- ^a^	+	- ^d^	-	-	-	-	-	-
Thm3	- ^a^	0.28 ± 0.03 ^a^	-	5.77 ± 0.26 ^g–l^	11.71 ± 0.03 ^d^	+	53.38 ± 0.25 ^a^	-	-	-	-	-	-
Thm6	3.07 ± 0.01 ^c–f^	0.26 ± 0.04 ^a^	-	5.64 ± 0.07 ^g–l^	18.65 ± 0.02 ^k^	+	- ^d^	-	-	-	-	-	-
Ab1	2.90 ± 0.18 ^cd^	0.21 ± 0.01 ^a^	-	5.54 ± 0.15 ^f–k^	25.75 ± 0.02 ^s^	-	- ^d^	-	-	-	-	-	-
Ab3	4.85 ± 0.33 ^m^	0.30 ± 0.01 ^a^	-	6.09 ± 0.23 ^j–l^	- ^a^	+++	49.23 ± 0.25 ^b^	-	-	-	-	-	-
Ab4	2.97 ± 0.20 ^cde^	0.27 ± 0.06 ^a^	-	5.90 ± 0.19 ^h–l^	2.25 ± 0.02 ^b^	-	- ^d^	-	-	-	-	-	-
Ab5	- ^a^	0.27 ± 0.07 ^a^	-	6.23 ± 0.10 ^klm^	15.06 ± 0.02 ^g^	-	- ^d^	-	-	-	-	-	-
Ab6	4.29 ± 0.22 ^j–m^	0.21 ± 0.01 ^a^	-	5.80 ± 0.14 ^g–l^	11.66 ± 0.04 ^d^	++	- ^d^	-	-	-	-	-	-
Of2	4.36 ± 0.46 ^klm^	0.31 ± 0.04 ^a^	-	6.32 ± 0.07 ^lm^	- ^a^	-	- ^d^	-	-	-	-	-	-
Ct2	2.59 ± 0.16 ^bc^	0.23 ± 0.00 ^a^	-	5.79 ± 0.13 ^g–l^	18.37 ± 0.04 ^i^	++	- ^d^	-	-	-	-	-	-
Ct4	4.53 ± 0.72 ^lm^	0.24 ± 0.00 ^a^	-	5.07 ± 0.15 ^efg^	26.03 ± 0.04 ^t^	++	- ^d^	-	-	-	-	-	-
Ct7	3.22 ± 0.03 ^c–g^	0.26 ± 0.09 ^a^	-	5.10 ± 0.19 ^efg^	22.68 ± 0.04 ^p^	-	- ^d^	-	-	-	-	-	-
Mz2	4.28 ± 0.24 ^j–m^	0.25 ± 0.03 ^a^	-	6.08 ± 0.27 ^j–l^	- ^a^	++	- ^d^	-	-	-	-	-	-
Mz4	3.68 ± 0.19 ^f–j^	0.27 ± 0.03 ^a^	-	5.75 ± 0.20 ^g–l^	22.12 ± 0.02 ^o^	-	- ^d^	-	-	-	-	-	-
Mz5	3.86 ± 0.17 ^h–k^	0.26 ± 0.03 ^a^	-	6.11 ± 0.10 ^j–l^	20.77 ± 0.02 ^m^	-	- ^d^	-	-	-	-	-	-
Pv1	3.99 ± 0.45 ^h–l^	0.27 ± 0.02 ^a^	-	5.43 ± 0.62 ^e–j^	27.88 ± 0.02 ^u^	-	- ^d^	-	-	-	-	-	-
Mcit7	4.11 ± 0.24 ^i–l^	0.25 ± 0.04 ^a^	-	4.26 ± 0.22 ^cd^	- ^a^	-	24.56 ± 0.50 ^c^	-	-	-	-	-	-

Values are presented as the mean ± SD of three independent replicates. Within each column, means followed by different lowercase letters differ significantly according to one-way analysis of variance (ANOVA), followed by Tukey’s HSD post hoc test (*p* < 0.05). Means sharing at least one letter are not significantly different. A dash (-) indicates that no activity was detected under the tested conditions.

**Table 2:** Antibiotic susceptibility profiles of the LAB strains to the seven tested antibiotics.

			Antibiotic				
Strain	DA2	E15	DO30	C30	AMP10	CN10	TE30
Levh7	I (17 ± 0.1)	S (31 ± 0.3)	I (18 ± 0.2)	S (32 ± 0.2)	S (43 ± 0.2)	R	I (18 ± 0.1)
Pl1	R (6 ± 0.1)	S (27 ± 0.3)	I (18 ± 0.2)	S (29 ± 0.2)	S (32 ± 0.3)	R	I (12 ± 0.1)
Pl5	R (8 ± 0.1)	S (27 ± 0.1)	S (23 ± 0.2)	S (31 ± 0.1)	S (39 ± 0.3)	R	S (18 ± 0.2)
JB3	I (15 ± 0.1)	S (30 ± 0.2)	S (25 ± 0.1)	S (31 ± 0.3)	S (28 ± 0.3)	R	I (15 ± 0.2)
JB4	I (16 ± 0.1)	S (31 ± 0.3)	S (30 ± 0.2)	S (32 ± 0.4)	S (33 ± 0.2)	R	I (19 ± 0.1)
L2	R (6 ± 0.1)	S (31 ± 0.1)	S (32 ± 0.3)	S (32 ± 0.2)	S (36 ± 0.3)	R	I (20 ± 0.1)
LB2	R (7 ± 0.3)	I (14 ± 0.2)	S (41 ± 0.5)	S (32 ± 0.3)	S (33 ± 0.1)	R	S (39 ± 0.3)
Lev1	R (8 ± 0.1)	S (27 ± 0.1)	S (21 ± 0.1)	S (31 ± 0.2)	S (33 ± 0.2)	R	I (13 ± 0.1)
Lev2	I (13 ± 0.2)	S (32 ± 0.2)	S (28 ± 0.3)	S (33 ± 0.1)	S (39 ± 0.1)	R	I (20 ± 0.3)
Lev5	R (8 ± 0.1)	S (27 ± 0.1)	S (23 ± 0.4)	S (26 ± 0.1)	S (29 ± 0.3)	R	I (17 ± 0.2)
Pmt4	R (10 ± 0.2)	S (24 ± 0.1)	S (19 ± 0.2)	S (26 ± 0.1)	S (27 ± 0.1)	R	I (17 ± 0.1)
Pmt5	R (11 ± 0.1)	S (27 ± 0.3)	S (23 ± 0.2)	S (29 ± 0.1)	S (30 ± 0.3)	R	I (16 ± 0.3)
Fg1	S (21 ± 0.3)	S (31 ± 0.2)	S (40 ± 0.3)	S (31 ± 0.1)	S (34 ± 0.4)	R	S (39 ± 0.4)
Fg8	R (7 ± 0.1)	S (33 ± 0.1)	S (25 ± 0.2)	S (32 ± 0.3)	S (43 ± 0.3)	R	S (25 ± 0.1)
Thm1	R (7 ± 0.2)	S (21 ± 0.1)	S (25 ± 0.3)	S (32 ± 0.2)	S (29 ± 0.4)	R	I (18 ± 0.1)
Thm2	I (15 ± 0.2)	S (30 ± 0.3)	S (24 ± 0.1)	S (29 ± 0.1)	S (40 ± 0.1)	R	S (17 ± 0.1)
Thm3	R (7 ± 0.1)	S (25 ± 0.1)	R (13 ± 0.4)	S (30 ± 0.3)	S (32 ± 0.2)	R	I (16 ± 0.1)
Thm6	R (9 ± 0.1)	S (31 ± 0.3)	S (21 ± 0.2)	S (33 ± 0.4)	S (32 ± 0.2)	R	S (17 ± 0.1)
Ab1	I (13 ± 0.1)	S (30 ± 0.2)	S (25 ± 0.3)	S (33 ± 0.3)	S (41 ± 0.5)	R	S (21 ± 0.1)
Ab3	I (17 ± 0.2)	S (27 ± 0.4)	S (22 ± 0.3)	S (32 ± 0.4)	S (39 ± 0.3)	R	I (18 ± 0.2)
Ab4	R (7 ± 0.1)	S (28 ± 0.2)	S (22 ± 0.3)	S (32 ± 0.3)	S (35 ± 0.2)	R	I (16 ± 0.1)
Ab5	I (18 ± 0.3)	S (35 ± 0.1)	S (30 ± 0.2)	S (35 ± 0.3)	S (45 ± 0.5)	R	S (21 ± 0.1)
Ab6	R (9 ± 0.2)	S (30 ± 0.2)	S (17 ± 0.1)	S (33 ± 0.3)	S (32 ± 0.2)	R	S (18 ± 0.3)
Of2	S (22 ± 0.2)	S (35 ± 0.4)	S (39 ± 0.2)	S (31 ± 0.4)	S (43 ± 0.5)	R	S (36 ± 0.2)
Ct2	S (20 ± 0.3)	S (30 ± 0.3)	S (35 ± 0.5)	S (32 ± 0.3)	S (40 ± 0.2)	R	S (34 ± 0.4)
Ct4	I (16 ± 0.2)	S (25 ± 0.3)	S (25 ± 0.1)	S (30 ± 0.4)	S (36 ± 0.3)	R	I (19 ± 0.3)
Ct7	I (20 ± 0.3)	S (28 ± 0.2)	S (21 ± 0.1)	S (31 ± 0.2)	S (37 ± 0.3)	R	S (22 ± 0.3)
Mz2	S (37 ± 0.4)	S (29 ± 0.4)	S (28 ± 0.2)	S (30 ± 0.2)	S (34 ± 0.3)	R	S (21 ± 0.2)
Mz4	I (15 ± 0.2)	S (29 ± 0.3)	S (24 ± 0.2)	S (31 ± 0.2)	S (41 ± 0.3)	R	I (18 ± 0.3)
Mz5	R (9 ± 0.1)	S (28 ± 0.2)	R (14 ± 0.3)	S (28 ± 0.4)	S (31 ± 0.3)	R	I (13 ± 0.1)
Pv1	I (20 ± 0.2)	S (30 ± 0.4)	S (23 ± 0.2)	S (31 ± 0.3)	S (41 ± 0.5)	R	S (20 ± 0.1)
Mcit7	S (24 ± 0.2)	S (31 ± 0.2)	S (38 ± 0.3)	S (33 ± 0.3)	S (37 ± 0.2)	R	S (33 ± 0.2)

R, resistant; I, intermediate; S, susceptible. Values in parentheses indicate inhibition-zone diameters in millimeters.

## Data Availability

The original contributions presented in this study are included in the article/Supplementary Material. Further inquiries can be directed to the corresponding authors.

## Supplementary Materials

The following supporting information can be downloaded at https://www.mdpi.com/article/10.3390/microorganisms14102282/s1, Table S1: Taxonomic identification and accession numbers of the different strains of the LAB collection used in this study; Table S2: Taxonomic identification and accession numbers of the four pathogens used in this study; Table S3: Annealing temperatures of the different lipopeptides- and bacteriocin-associated primers; Table S4: LAB standards for Antibiotic Breakpoints according to EFSA.

## Abbreviations

The following abbreviations are used in this manuscript:

LAB	Lactic acid bacteria
PGPR	Plant-growth-promoting rhizobacteria
ANOVA	Analysis of variance
AT	AT minimal medium/broth*
CAS	Chrome azurol S
CFU	Colony-forming units
CNRST	Center for Scientific and Technical Research
DAS	Data Availability Statement
DNA	Deoxyribonucleic acid
DNase	Deoxyribonuclease
dNTP	Deoxyribonucleoside triphosphate
ELISA	Enzyme-linked immunosorbent assay
FDA	Food and Drug Administration
GRAS	Generally Recognized as Safe
HCN	Hydrogen cyanide
HDTMA	Hexadecyltrimethylammonium bromide
HSD	Honestly significant difference
IAA	Indole-3-acetic acid
IR	Inhibition rate
MRS	de Man, Rogosa, and Sharpe
PCA	Principal component analysis
PC1	Principal component 1
PC2	Principal component 2
PCR	Polymerase chain reaction
PDA	Potato dextrose agar
PGP	Plant-growth-promoting/plant growth promotion
PGPI	Plant growth promotion index
PGPMs	Plant-growth-promoting microorganisms
PBS	Phosphate-buffered saline
pNPP	p-Nitrophenyl phosphate
PSU	Percent siderophore units
QPS	Qualified Presumption of Safety
SD	Standard deviation
SDW	Sterile distilled water
SMA	Skim milk agar
SPSS	Statistical Package for the Social Sciences
UM6P	Mohammed VI Polytechnic University
