# Supplementary material for: Potential of Lactic Acid Bacteria as Plant-Growth-Promoting Bioinoculant Candidates with In Vitro Antifungal Activity: Insights from Brassica napus as a Model System
Source: Microorganisms. 2026 Oct 7;14(10):2282. doi: 10.3390/microorganisms14102282 (PMC13707870; doi:10.3390/microorganisms14102282)
Supplement: Supplementary file 1 [file microorganisms-14-02282-s001.zip › microorganisms-4590925-supplementary.pdf]

Table S1: Taxonomic identification and accession numbers of the different strains of the LAB collection used in this study.

**strains**	**species**	**Accession number**	**Isolation source**
Thm1	*Lactiplantibacillus plantarum*	PV930011	Soil
Pmt5	*Lactiplantibacillus plantarum*	PV930012	Soil
Pl5	*Lactiplantibacillus pentosus*	PV930013	Fruit surface
CT4	*Lactiplantibacillus plantarum*	PV930014	Soil
Pl1	*Lactiplantibacillus plantarum*	PV930015	Fruit surface
Thm2	*Lactiplantibacillus plantarum*	PV930016	Soil
Ab3	*Levilactobacillus brevis*	PV930017	Soil
JB3	*Lactiplantibacillus plantarum*	PV930018	Fresh cheese
Thm3	*Lactiplantibacillus plantarum*	PV930019	Soil
JB4	*Enterococcus durans*	PV930020	Fresh cheese
Pmt4	*Levilactobacillus brevis*	PV930021	Soil
Fg1	*Lactiplantibacillus plantarum*	PV930022	Soil
Lev5	*Levilactobacillus brevis*	PV930023	Sourdough
Lev2	*Lactiplantibacillus plantarum*	PV930024	Sourdough
LevH7	*Levilactobacillus brevis*	PV930025	Sourdough
Lev1	*Lactiplantibacillus plantarum*	PV930026	Sourdough
AB5	*Levilactobacillus brevis*	PV930027	Soil
Ct2	*Levilactobacillus brevis*	PV930028	Soil
Mz5	*Lactiplantibacillus plantarum*	PV930029	Soil
Mcit7	*Lactiplantibacillus plantarum*	PV930030	Honey
L2	*Lactiplantibacillus plantarum*	PV930031	Fresh Cow Milk
Of2	*Lacticaseibacillus rhamnosus*	PV930032	Fermented olives
Ab4	*Levilactobacillus brevis*	PV930033	soil
LB2	*Levilactobacillus brevis*	PV930034	Fermented milk
Pv1	*Lactiplantibacillus plantarum*	PV930035	Vegetable surface
Mz4	*Lactiplantibacillus plantarum*	PV930036	Soil
Mz2	*Lactiplantibacillus plantarum*	PV930037	Soil
Thm6	*Lactiplantibacillus plantarum*	PV930038	Soil
Ct7	*Lactiplantibacillus plantarum*	PV930039	Soil
Fg8	*Lactiplantibacillus plantarum*	PV930040	Soil
Ab6	*Lactiplantibacillus pentosus*	PV930041	Soil
Ab1	*Lactiplantibacillus plantarum*	PV930042	Soil

Table S2: Taxonomic identification and accession numbers of the four pathogens used in this study.

Strain	Species	Accession number	Isolation source
FSS6	*Fusarium equiseti*	OM876903	Beetroot
PR1	*Botrytis cinerea*	OQ691642	Apple
GK	*Geotrichum citri-aurantii*	PQ858624	Orange
PB1	*Pythium heterothallicum (globisporongium heterothallicum)*	QB114390	Beetroot

Table S3. Annealing temperatures of the different lipopeptides- and bacteriocin- associated primers

Antimicrobial compound	Primer pair	Sequence (5'-3')	Target gene / locus	Expected band size	Annealing T°C	Reference
Bacillomycin	BACC1F/ BACC1R	BACC1F 5'-GAAGGACACGGCAGAGAGTC-3' BACC1R 5'-CGCTGATGACTGTTCATGCT-3'	Bacillomycin biosynthetic gene	875 bp	60 °C	[1,2]
Fengycin	FEND1F/ FEND1R	FEND1F 5'-TTTGGCAGCAGGAGAAGTTT-3' FEND1R 5'-GCTGTCCGTTCTGCTTTTTC-3'	fenD	964 bp	62 °C	[1,2]
Iturin	ITUP1F/ ITUP1R	ITUP1F 5'-AGCTTAGGGAACAATTGTCATCGGGGCTTC-3' ITUP2R 5'-TCAGATAGGCCGCCATATCGGAATGATTCG-3'	Iturin biosynthetic gene cluster	≈ 2 kb	45 °C	[2]
Surfactin	P17/ P18	P17: 5'-ATGAAGATTTACGGAATTTA-3' and P18: 5'-TTATAAAAGCTCTTCGTACG-3'	sfp	675 bp	53 °C	[2,3]
Subtiline	SpaS_F/SpaS_R	spaS-F 5'-TGTCATGGTTACAGCGGTATCGGTC-3' spaS-R 5'-AGTGCAAGGAGTCAGAGCAAGGTGA-3'	sboA / subtilosin locus	1220 bp	58 °C	[4]
Subtilosin	oSboP1N/ oSboP2N	OsboP1N 5'-CCTCATGACCAGGACTTCGCCTT-3'OsboP2N 5'-CGGTGCCGAGCGCTTCAGGT-3'	spaS	152 bp	58 °C	[4]

Table S4. LAB standards for Antibiotic Breakpoints according to EFSA

Antibiotic (Code)	Disk Potency (µg)	Resistant (≤ mm)	Intermediate (mm)	Susceptible (≥ mm)	Reference
Clindamycin (DA 2)	2	12	13-19	20	EFSA, ISO 10932
Erythromycin (E15)	15	13	14-20	21	EFSA, ISO 10932
Doxycycline (DO30)	30	14	15-22	23	EFSA, ISO 10932
Chloramphenicol (C30)	30	13	14-22	23	EFSA, ISO 10932
Ampicillin (AMP 10)	10	14	15-21	22	EFSA, ISO 10932
Gentamicin (CN 10)	10	Intrinsic Resistance	-	-	EFSA, ISO 10932
Tetracycline (TE 30)	30	12	13-20	21	EFSA, ISO 10932
